# A Bacteriophage-Based, Highly Efficacious, Needle- and Adjuvant-Free, Mucosal COVID-19 Vaccine

**DOI:** 10.1128/mbio.01822-22

**Published:** 2022-07-28

**Authors:** Jingen Zhu, Swati Jain, Jian Sha, Himanshu Batra, Neeti Ananthaswamy, Paul B. Kilgore, Emily K. Hendrix, Yashoda M. Hosakote, Xiaorong Wu, Juan P. Olano, Adeyemi Kayode, Cristi L. Galindo, Simran Banga, Aleksandra Drelich, Vivian Tat, Chien-Te K. Tseng, Ashok K. Chopra, Venigalla B. Rao

**Affiliations:** a Bacteriophage Medical Research Center, Department of Biology, The Catholic University of America, Washington, DC, USA; b Department of Microbiology and Immunology, University of Texas Medical Branch, Galveston, Texas, USA; c Department of Pathology, University of Texas Medical Branch, Galveston, Texas, USA; d Center for Biodefense and Emerging Infectious Diseases, University of Texas Medical Branch, Galveston, Texas, USA; e Sealy Institute for Vaccine Sciences, University of Texas Medical Branch, Galveston, Texas, USA; f Institute for Human Infections and Immunity, University of Texas Medical Branch, Galveston, Texas, USA; g Department of Biology, Western Kentucky University, Bowling Green, Kentucky, USA; University of Pittsburgh

**Keywords:** needle-free intranasal vaccine, bacteriophage T4 mucosal platform, bacteriophage CRISPR engineering, broad immunity to SARS-CoV-2 variants, sterilizing immunity

## Abstract

The U.S. Food and Drug Administration-authorized mRNA- and adenovirus-based SARS-CoV-2 vaccines are intramuscularly injected in two doses and effective in preventing COVID-19, but they do not induce efficient mucosal immunity or prevent viral transmission. Here, we report the first noninfectious, bacteriophage T4-based, multicomponent, needle- and adjuvant-free, mucosal vaccine harboring engineered Spike trimers on capsid exterior and nucleocapsid protein in the interior. Intranasal administration of two doses of this T4 SARS-CoV-2 vaccine 21 days apart induced robust mucosal immunity, in addition to strong systemic humoral and cellular immune responses. The intranasal vaccine induced broad virus neutralization antibody titers against multiple variants, Th1-biased cytokine responses, strong CD4^+^ and CD8^+^ T cell immunity, and high secretory IgA titers in sera and bronchoalveolar lavage specimens from vaccinated mice. All of these responses were much stronger in intranasally vaccinated mice than those induced by the injected vaccine. Furthermore, the nasal vaccine provided complete protection and sterilizing immunity against the mouse-adapted SARS-CoV-2 MA10 strain, the ancestral WA-1/2020 strain, and the most lethal Delta variant in both BALB/c and human angiotensin converting enzyme (hACE2) knock-in transgenic mouse models. In addition, the vaccine elicited virus-neutralizing antibodies against SARS-CoV-2 variants in bronchoalveolar lavage specimens, did not affect the gut microbiota, exhibited minimal lung lesions in vaccinated and challenged mice, and is completely stable at ambient temperature. This modular, needle-free, phage T4 mucosal vaccine delivery platform is therefore an excellent candidate for designing efficacious mucosal vaccines against other respiratory infections and for emergency preparedness against emerging epidemic and pandemic pathogens.

## INTRODUCTION

The mRNA, adenovirus-based, and inactivated viral vaccines currently used for human immunization are having a tremendous impact on tamping down the devastating COVID-19 pandemic that has caused millions of deaths across the globe. Administered by intramuscular injections, these vaccines remain as the major source for the rest of the world’s unvaccinated population (1). However, there are still no needle-free mucosal vaccines authorized for human administration (2, 3).

Although the injectable vaccines are highly effective (70 to 95%) in preventing severe symptoms of the disease, hospitalization of patients, and deaths, these vaccines do not efficiently prevent viral acquisition or viral shedding from infected individuals. This is attributed to the lack of vaccine-induced secretory IgA (sIgA) mucosal immune responses in the respiratory airways that could prevent person-to-person transmission (3–6). Therefore, risk of transmission from vaccinated subjects, who are susceptible to SARS-CoV-2 infection, as seen currently on a global scale with the highly transmissible Omicron variants, remains a serious concern (7).

The current vaccines developed using the Spike protein of the ancestral SARS-CoV-2 strain (Wuhan-Hu-1) show progressively diminished efficacy against the subsequently emerged viral variants of concern (VOCs) such as Alpha, Beta, Gamma, Delta, and—most recently—Omicron and its subvariants, which are more efficiently transmitted and/or more lethal. The evolutionary space for emergence of newer SARS-CoV-2 variants/subvariants that are even more efficiently transmissible and also more lethal that might render the current vaccines ineffective remains a worrisome and real possibility (8).

Considering the evolutionary path of the virus, the most desired next-generation vaccine(s) would be one that can induce strong mucosal immunity, in addition to broader systemic immunity (2, 3, 9–11). Elicitation of target-specific mucosal antibodies at the portal of virus entry would block virus acquisition, as well as shedding of infectious virus particles and their potential transmission (11–19). Such platforms are of particular strategic importance at this stage of the COVID-19 pandemic. In addition, platforms that are needle- and adjuvant-free and stable at ambient temperatures would greatly accelerate global distribution efforts, not only for controlling the current COVID-19 pandemic but also for any future epidemic or pandemic. Furthermore, needle-free vaccines can be administered easily and safely and may provide the best option to vaccinate children.

**FIG 1:**
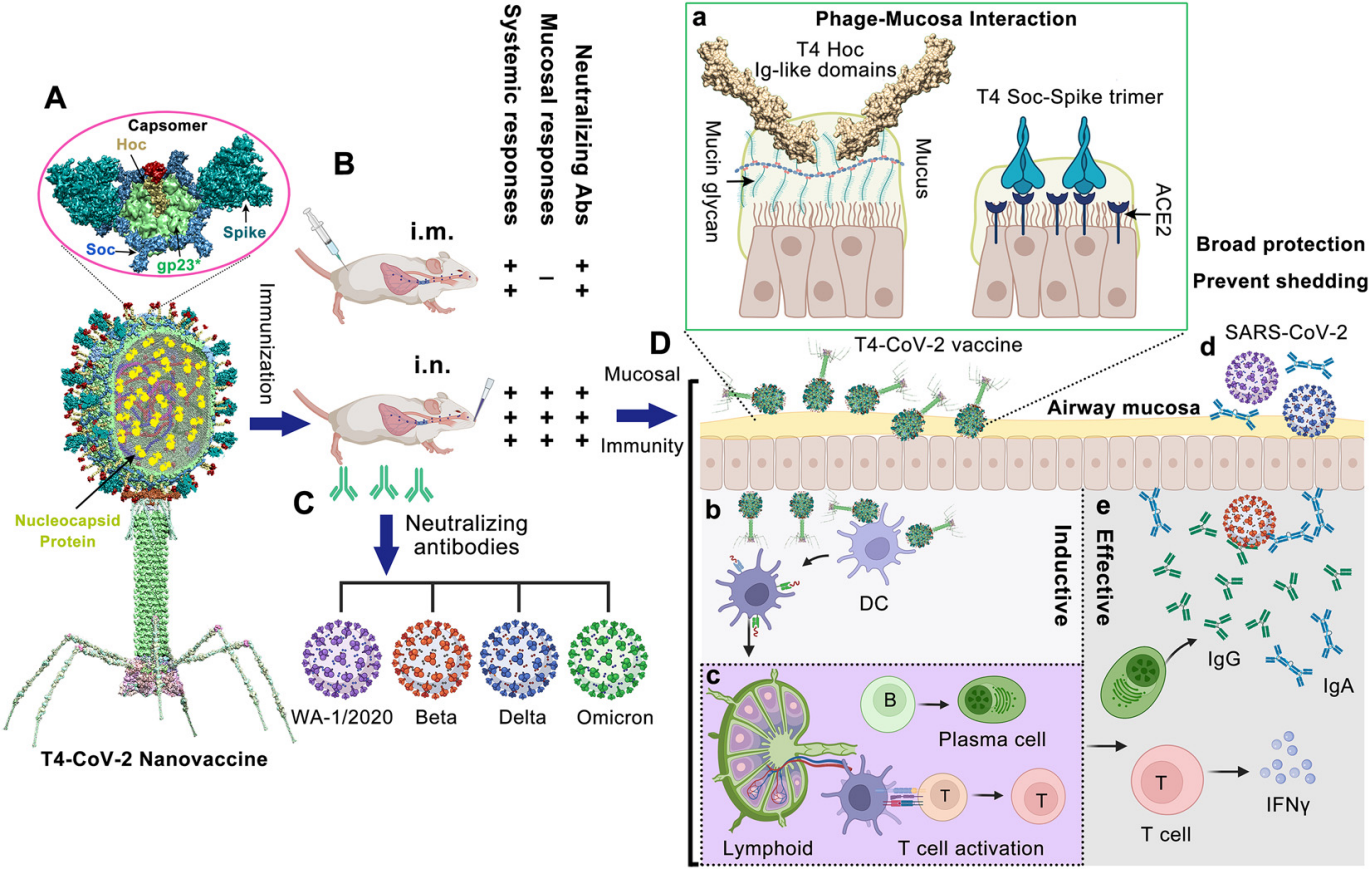
Intranasal vaccination of mice using bacteriophage T4-CoV-2 vaccine and possible mechanisms of protection. (A) Structural model of T4-CoV-2 nanovaccine constructed by CRISPR engineering (20). The enlarged view shows a single hexameric capsomer consisting of six subunits of major capsid protein gp23* (green), trimers of Soc (blue), and a Hoc fiber (yellow) at the center of capsomer. The NP, Ee, and SpyCatcher gene were “hard-wired” by inserting the respective expressible genes into phage genome, which resulted in display of Ee peptide (red, 155 copies per T4) at the tip of Hoc fiber, SpyCatcher as Soc fusion on capsid surface (~200 copies per capsid), and packaging of NP molecules (yellow, 100 copies per T4) inside the capsid. The Spy-tagged Spike trimer (cyan) purified from ExpiCHO cells was then conjugated to Soc-SpyCatcher (89). (B and C) Comparison between i.m. and i.n. T4-CoV-2 vaccination (B) and the elicited neutralizing antibodies against SARS-CoV-2 and its VOCs, including Beta, Delta, and Omicron (C). (D) Mucosal immune responses induced by T4-CoV-2 i.n. vaccination. (a) After i.n. inoculation, T4-CoV-2 particles would bind to mucosal cells (i) through the Ig-like domains of Hoc fibers which interact with mucin glycoproteins and (ii) through the displayed S-trimers which bind to ACE2 that is abundant in nasal epithelium. (b and c) Next, the antigen-presenting cells in the respiratory tract, such as dendritic cells (DC) capture T4-CoV-2 phage (b), migrate to mucosa-associated lymphoid tissues, and present the antigens to lymphocytes, including B and T cells (c). (d and e) The activated B cells become plasma cells secreting anti-SARS-CoV-2 IgG and IgA, which neutralize virus within the respiratory tract. The activated T cells migrate to lungs, produce cytokines and regulate the immune responses, and/or directly attack virus-infected host cells. These mucosal immune responses produced by T4-CoV-2 i.n. vaccination might be able to block viral entry (host’s viral acquisition) and viral exit (host’s viral shedding) in the respiratory tract.

We recently reported (20) the development of a “universal” phage T4 vaccine design platform (Fig. 1A) by Clustered Regularly Interspaced Short Palindromic Repeats (CRISPR) engineering (21, 22) that can rapidly generate multivalent vaccine candidates. Of the candidates screened, an optimal COVID-19 vaccine was selected. It consisted of T4 phage decorated with ~100 copies of prefusion-stabilized Spike ectodomain trimers (S-trimers) on the surface of 120 × 86-nm virus capsid (Fig. 1A). In addition, the vaccine also contained SARS-CoV-2 nucleocapsid protein (NP) packaged in the capsid core and a 12-amino-acid peptide of the putative external domain of E protein (Ee) fused to the highly antigenic outer capsid protein (Hoc) displayed on the capsid surface (Fig. 1A). This vaccine candidate (referred to as T4-CoV-2) elicited robust immunogenicity, virus neutralizing activity, and complete protection against ancestral SARS-CoV-2 challenge in a mouse model (20).

The protective immunity of the T4-CoV-2 nanovaccine could potentially be because of the repetitive and symmetrical arrays of S-trimers on phage particles, resembling the PAMPs (pathogen-associated molecular patterns) present on human viral pathogens (23–26). This architecture might mimic, in some respects, the spikes displayed on the SARS-CoV-2 virion (27). Therefore, we hypothesized that it is probable that such a T4-CoV-2 nanoparticle when exposed to nasal mucosal surfaces might be recognized as a natural viral intruder by the resident immune cells, stimulating strong mucosal as well as systemic immune responses (Fig. 1B to D). Furthermore, the S-trimer-displayed T4-CoV-2 nanoparticle could efficiently bind to the nasal epithelium that has the highest concentration of angiotensin-converting enzyme 2 (ACE2) receptors (28). In addition, the 155 symmetrically arranged Ig-like Hoc fibers on the T4 capsid are reported to interact with mucin glycoproteins, potentially capturing the T4-CoV-2 vaccine particles at the nasal mucosa (29, 30) (Fig. 1Da), leading to translocation across the epithelial layer (31) and uptake by antigen-presenting cells (32).

Here, we tested this hypothesis in a mouse model by intranasal (i.n.) inoculation of the T4-CoV-2 vaccine and performed exhaustive immunological analyses. Remarkably, this needle- and adjuvant-free vaccination with noninfectious T4-CoV-2 nanoparticles induced strong mucosal, humoral, and cellular immunity. The responses included Spike-specific CD4^+^ helper and effector T cells and CD8^+^ killer T cells and broad neutralization of SARS-CoV-2 VOCs, including B.1.135 Beta, B.1.617.2 Delta, and B.1.1.529 Omicron, in both BALB/c and human ACE2 (hACE2) transgenic mouse models. These responses elicited by needle-free vaccination are much stronger than that elicited by the injected vaccine, and secretory IgA antibodies were measured only in i.n.-vaccinated mice. Furthermore, the T4-CoV-2 vaccine is stable at ambient temperature, which can be easily manufactured and distributed at a modest cost. Thus, this noninfectious phage-based mucosal vaccine is an excellent candidate for boosting the immunity of immunized individuals and/or as a next-generation COVID vaccine for the unimmunized populations.

## RESULTS

### Needle-free T4-CoV-2 nanovaccine stimulates robust humoral and cellular immune responses against SARS-CoV-2 and VOCs.

**FIG 2:**
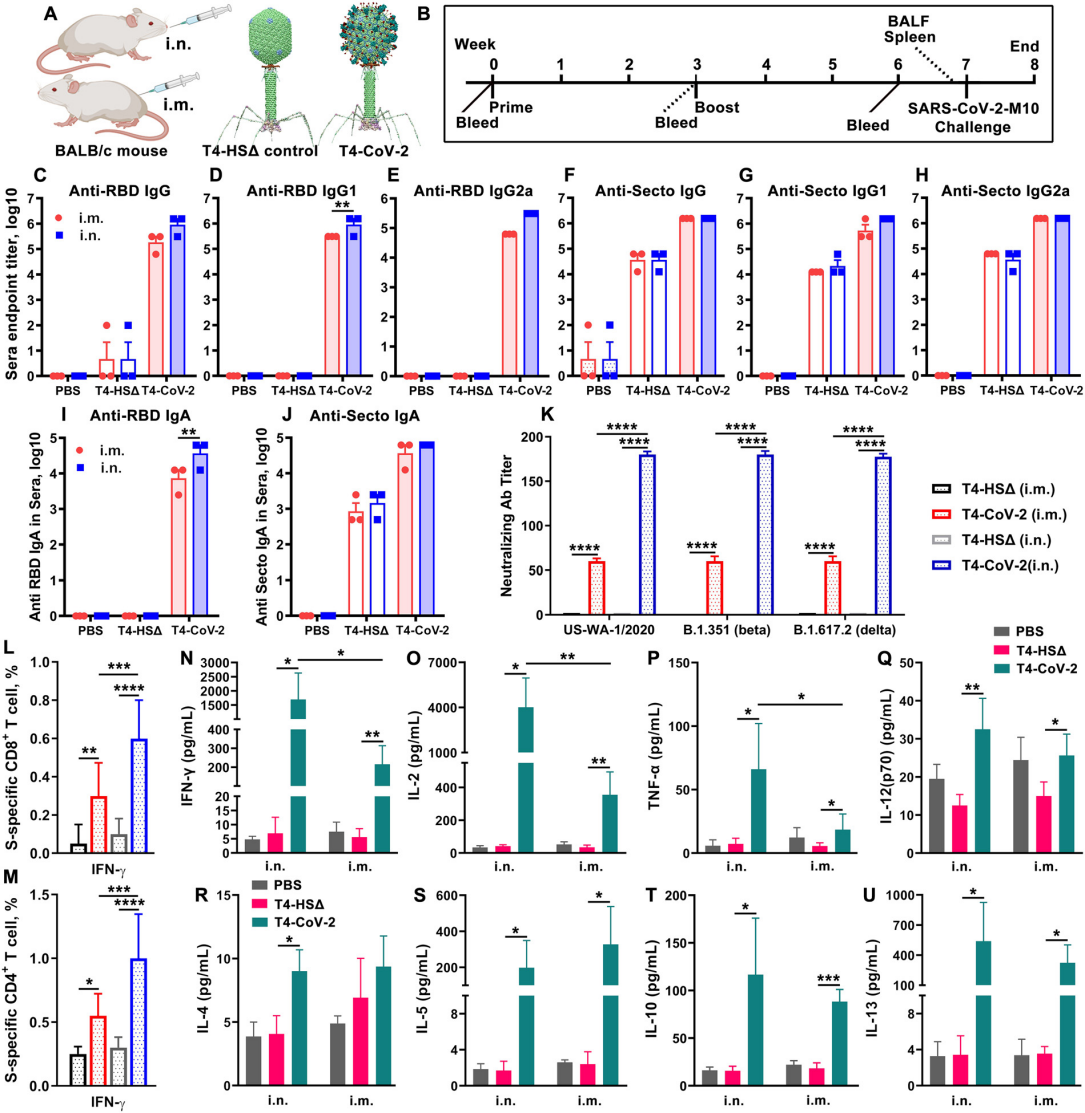
Intranasal immunization elicited greater anti-Spike/RBD systemic humoral and cellular responses over intramuscular immunization. (A) Schematic of T4-CoV-2 i.n. and i.m. vaccinations with T4-HocΔ-SocΔ (T4-HSΔ, lacking bot Hoc and Soc) phage (left, vector control) and T4-CoV-2 recombinant phage (right, vaccine phage). T4-CoV-2 recombinant phage was constructed usingT4-HSΔ as a scaffold. (B) Scheme for vaccination and challenge. (C to J) Antibody responses in sera of immunized mice at day 21 after the last dose. ELISA was used to measure reciprocal endpoint antibody titers of anti-RBD IgG (C), anti-RBD IgG1 (D), anti-RBD IgG2a (E), anti-Secto IgG (F), anti-Secto IgG1 (G), anti-Secto IgG2a (H), anti-RBD IgA (I), and anti-Secto IgA (J). Data represent means ± the standard errors of the mean (SEM). The data are from three pooled independent experiments (*n* = 22 for T4-CoV-2, *n* = 10 for T4-HSΔ, and *n* = 5 for PBS). (K) The virus neutralizing activity in sera of i.m.- and i.n.-immunized mice was determined by a Vero E6 cell cytopathic assay using ancestral SARS-CoV-2 US-WA-1/2020, B.1.351 (Beta), and B.1.617.2 (Delta) strains. (L and M) Cellular immune responses. The percentages of IFN-γ^+^ CD8^+^ (L) and IFN-γ^+^ CD4^+^ (M) cells were plotted. Panels L and M use the same color coding as panel K. (N to U) Cytokine responses. Representative Th1 (N to Q) and Th2 (R to U) cytokines are shown. For panels K to M, two-way ANOVA with Tukey *post hoc* test was used to compare multiple groups. For panels N to U, a nonparametric Student *t* test was used to compare T4-vector control versus T4-CoV-2 vaccine groups and i.n. versus i.m. routes of vaccination. *, *P* < 0.05; **, *P* < 0.01; ***, *P* < 0.001; ****, *P* < 0.0001. Data represent means ± the standard deviations and are representative of five biological replicates.

The immunogenicity of the T4-CoV-2 nanovaccine was first evaluated in 5-week-old wild-type (WT) BALB/c mice. In a standard prime-boost regimen (Fig. 2A and B), animals received two intramuscular (i.m.) or i.n. doses of either the T4-HocΔ-SocΔ phage (T4-HSΔ; T4 vector control lacking Hoc, Soc, or any SARS-CoV-2 antigens) or the T4-CoV-2 phage vaccine decorated with 20 μg (high dose; ~2.5 × 10^11^ particles), 4.8 μg (medium dose, ~6 × 10^10^ particles), or 0.8 μg (low dose; ~1 × 10^10^ particles) of SARS-CoV-2 Spike ectodomain (Secto; amino acids 1 to 1213) trimers. In a one-dose regimen, animals received a single i.m. high-dose of the T4-CoV-2 vaccine.

**(i) Antibody responses (IgG, isotypes, and IgA).** To evaluate humoral antibody responses, sera were collected on day 21 after the last dose (Fig. 2B), and IgG, IgG1, and IgG2a antibodies specific to the Secto protein or the receptor-binding domain (RBD) were quantified by enzyme-linked immunosorbent assay (ELISA; Fig. 2C to H; see also Fig. S1 in the supplemental material). The phosphate-buffered saline (PBS) and T4-vector control groups, as expected, induced no significant antigen-specific antibodies, whereas the T4-CoV-2-vaccinated groups (either i.m. or i.n.) triggered high levels of IgG antibodies (endpoint titers up to 312,500) (Fig. 2C and F).

10.1128/mbio.01822-22.1 FIG S1 Anti-Spike/RBD systemic humoral responses in sera from i.m.- or i.n.-administered BALB/c mice using various doses of T4-CoV-2 vaccine. ELISA assays were performed to measure reciprocal endpoint antibody titers of sera from i.m. (black) or i.n. (red) vaccinations: anti-RBD IgG (A), anti-RBD IgG1 (B), anti-RBD IgG2a (C), anti-RBD IgA (D), anti-Secto IgG (E), anti-Secto IgG1 (F), anti-Secto IgG2a (G), and anti-Secto IgA (H). PBS and T4-HSΔ were used as naive and vector controls, respectively. SD, single-dose. Data represent the means ± the SEM. The data are from three pooled independent experiments (*n* = 22 for T4-CoV-2, *n* = 10 for T4-HSΔ, and *n* = 5 for PBS). *P* values were calculated using ANOVA with Tukey *post hoc* test to compare multiple groups. *, *P* < 0. 1; **, *P* < 0.01; ***, *P* < 0.001. Download FIG S1, TIF file, 2.8 MB. Copyright © 2022 Zhu et al. 2022 Zhu et al. https://creativecommons.org/licenses/by/4.0/ This content is distributed under the terms of the Creative Commons Attribution 4.0 International license.

High levels of both Th1 (IgG2a) and Th2 (IgG1) subtype antibodies (endpoint titers up to 312,500) were induced by i.m. and i.n. immunizations, demonstrating that the T4-CoV-2 vaccine triggered balanced Th1- and Th2-derived antibody responses (Fig. 2D, E, G, and H) compared to the alum-adjuvanted subunit vaccines that showed strong Th2-bias (20). The balanced immune response was also uniformly recapitulated in a dose response experiment. Nearly the same levels of Th1 and Th2 antibody responses were elicited with the medium dose as with the high dose, while the levels were lower (5 to 25-fold) with the low-dose or single-dose antigen (see Fig. S1).

Intriguingly, the T4-CoV-2 vaccine induced high levels of Spike-specific serum IgA antibodies (endpoint titers up to 62,500) when administered by either the i.m. or the i.n. route (Fig. 2I and J). This is notable because IgA stimulation is not commonly observed in traditional vaccines, including the current COVID-19 vaccines. For example, the adenovirus-based vaccines do not elicit significant Spike-specific serum IgA titers when injected i.m. (15). Elicitation of serum IgA is considered desirable for an effective COVID-19 vaccine because IgA antibodies are reported to have anti-inflammatory activity and are more potent than IgG in neutralizing SARS-CoV-2 virus during the early phase of infection (14).

**(ii) Virus-neutralizing antibodies.** To further analyze humoral immunity, the virus-neutralizing activity of the elicited antibodies was determined by Vero E6 cell cytopathic assay using the SARS-CoV-2 WA-1/2020 ancestral strain in the United States (33). As shown in Fig. S2A, the T4-CoV-2 vaccine induced strong neutralizing activity in the sera of all immunized mice. Significantly higher neutralizing antibody titers were detected in mice immunized i.m. with two doses of the T4-CoV-2 vaccine than with a single-dose immunization (see Fig. S2A). Importantly, a higher neutralizing antibody titer (3-fold) was induced by i.n. vaccination compared to i.m. high-dose immunization (see Fig. S2A).

10.1128/mbio.01822-22.2 FIG S2 Neutralizing antibody and cellular immune responses in i.m.- and i.n.-vaccinated BALB/c mice. (A) Virus neutralizing activity in sera of i.m.- and i.n.-vaccinated mice was determined by Vero E6 cell cytopathic assay using ancestral SARS-CoV-2 US-WA-1/2020 strain. (B and C) Cellular immune responses after stimulation with purified Secto trimers. Cells were stained with T cell surface markers CD3, CD4, and CD8, followed by intracellular TNF-α and IL-17A staining. The percentages of TNF-α^+^ CD8^+^ (B), IL-17A^+^ CD8^+^ (B), TNF-α^+^ CD4^+^ (C), and IL-17A^+^ CD4^+^ (C) cells were plotted. (D and E) Cellular immune responses after stimulation with S and NP peptides. The percentages of IFN-γ^+^, TNF-α^+^, or IL-17A^+^ cells in CD8^+^ (D) or CD4^+^ (E) cells were plotted. (F and G) T cell proliferation in mice immunized with T4-CoV-2 vaccine. Spleens were harvested from mice 21 days after the boost. Splenocytes were isolated and stimulated with either purified S-protein trimer (F) or S and NP peptides (G). Cells were stained for T cell surface marker CD3, as well as for incorporated BrdU, and then analyzed by flow cytometry. The percentage of BrdU incorporation in CD3^+^ cells was plotted. (H to N) Splenocyte cytokine response to S and NP peptide stimulation. The cytokines in cell culture supernatants were analyzed by using a Bioplex-23 assay. Representative Th1 (H to K) and Th2 (L to N) cytokines are shown. For panels A to G, data represent the means ± the standard deviations of five biological replicates. Two-way ANOVA with Tukey *post hoc* test was used (*, *P* < 0.05; **, *P* < 0.01; ***, *P* < 0.001; ****, *P* < 0.0001). For panels H to N, statistical significance was determined using a nonparametric Student *t* test comparing to T4-HSΔ vector versus T4-CoV-2 groups and i.n. versus i.m. administration groups (*, *P* < 0.05; **, *P* < 0.01). Data represent the means ± the standard deviations of five biological replicates. Download FIG S2, TIF file, 2.8 MB. Copyright © 2022 Zhu et al. 2022 Zhu et al. https://creativecommons.org/licenses/by/4.0/ This content is distributed under the terms of the Creative Commons Attribution 4.0 International license.

It is well known that the Beta and Delta variants escape vaccine-induced immune responses (34). Intriguingly, the T4-CoV-2 vaccine elicited comparable virus neutralizing activities to WA-1/2020, Beta (B.1.351), and Delta (B.1.617.2) VOCs (Fig. 1K). In addition, an ~3-fold-higher neutralizing antibody titer against SARS-CoV-2 and its VOCs was elicited by i.n. vaccination of mice compared to i.m. immunization, while no detectable neutralizing activity was detected in T4 vector or PBS control groups (Fig. 1K).

**(iii) Cell-mediated immunity.** To evaluate cellular immune responses, splenocytes were harvested from mice on day 26 after the boost (Fig. 1B). Antigen-specific CD8^+^ and CD4^+^ T cells were identified after *ex vivo* restimulation with either S-trimer (Fig. 2L and M; see also Fig. S2B and C) or with SARS-CoV-2 peptides spanning the S and NP proteins (see Fig. S2D and E). The samples were then analyzed by intracellular staining of accumulated cytokines and flow cytometry. The percentages of CD8^+^ and CD4^+^ T cells positive for interferon gamma (IFN-γ) (Fig. 2L and M), tumor necrosis factor alpha (TNF-α; see Fig. S2B to E), or interleukin-17A (IL-17A; see Fig. S2B to E) were elevated in T4-CoV-2-immunized mice compared to the T4 vector control group irrespective of the immunization routes and the virus-specific stimulants used.

IFN-γ is a predominant cytokine secreted by effector CD8^+^ T cells, Th1 CD4^+^ T cells, and NK cells (35). More specifically, with restimulation of splenocytes using S protein, significant levels of IFN-γ^+^ CD8^+^ cells, which play a critical role in SARS-CoV-2 viral clearance, were observed in i.n.-immunized mice (0.6%, *P* < 0.0001 compared to the vector control) (Fig. 2L). In addition, significantly elevated percentages of CD4^+^ T cells producing IFN-γ were detected in the i.n. group (1%) in comparison to the i.m. group (0.55%) of vaccinated mice (*P* < 0.001 between i.n. and i.m.) (Fig. 2M; see also Fig. S2C). These data indicated an enhanced Th1-mediated immunity induced by i.n. administration of the vaccine. Of note, we did not observe significant differences between i.n. and i.m. routes of immunization regarding either the IFN-γ^+^ CD8^+^ cells or the IFN-γ^+^ CD4^+^ cells when restimulated with S and NP peptides (see Fig. S2D and E). Probably, the conformational epitopes in S and NP proteins could contribute to these higher IFN-γ level differences in the i.n. group of animals. The robust T cell cytokine responses paralleled greater T cell proliferation in both i.n.- and i.m.-immunized groups of animals compared to the T4 vector control group (see Fig. S2F and G).

In addition, representative Th1 and Th2 cytokines in cell supernatants of the splenocytes were analyzed by the Bio-Plex platform. Both routes of immunization triggered increased production of Th1 cytokines (IFN-γ, IL-2, TNF-α, and IL-12-p70) (Fig. 2N to Q; see also Fig. S2H to K) and Th2 cytokines (IL-4, IL-5, IL-10, and IL-13) (Fig. 2R to U; see also Fig. S2L to N) compared to controls when splenocytes were stimulated with S-trimer (Fig. 2N to U) or S and NP peptides (see Fig. S2H to N). Increases in Th1 and Th2 cytokine levels by T4-CoV-2 immunization were consistent with induction of balanced Th1 and Th2 antibodies and cellular immune responses, as described above. Importantly, the levels of the main Th1 cytokines, including IFN-γ (*P* < 0.05), IL-2 (*P* < 0.01), and TNF-α (*P* < 0.05), were significantly higher in animals immunized by the i.n. route than those in mice immunized i.m. (Fig. 2N to P; see also Fig. S2H to J). These data indicated that T4-CoV-2 i.n. immunization most likely produced more Th1-biased immune responses compared to i.m. immunization. The vaccine-associated enhanced respiratory disease has not usually occurred when strong Th1 cell responses are induced. Therefore, considering that the COVID-19 vaccine designs developed to date have attempted to elicit either a Th1-biased or a Th1/Th2-balanced cell response (36, 37), the T4-CoV-2 vaccine generated the desirable responses.

### Needle-free T4-CoV-2 vaccination elicits robust mucosal immune responses.

**FIG 3:**
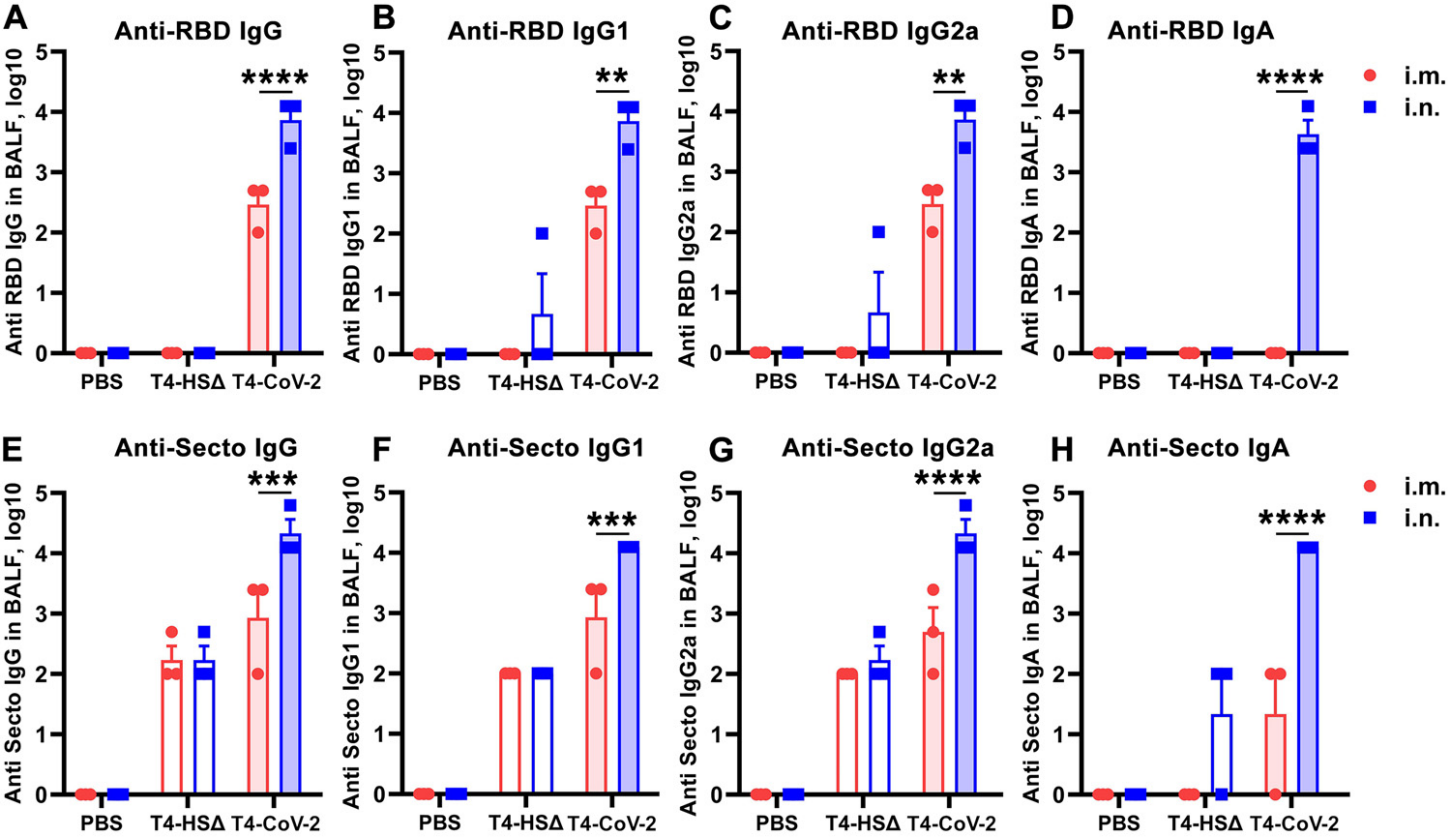
Intranasal immunization with T4-CoV-2 vaccine induced robust mucosal immune responses. The reciprocal endpoint antibody titers in BALF of anti-RBD IgG (A), anti-RBD IgG1 (B), anti-RBD IgG2a (C), anti-RBD IgA (D), anti-Secto IgG (E), anti-Secto IgG1 (F), anti-Secto IgG2a (G), and anti-Secto IgA (H) are shown. Data represent means ± the SEM. The data are from three pooled independent experiments (*n* = 12 for T4-CoV-2, *n* = 10 for T4-HSΔ, and *n* = 5 for PBS). The titers between i.m. and i.n. route were compared and statistically analyzed by two-way ANOVA (**, *P* < 0.01; ***, *P* < 0.001; ****, *P* < 0.0001).

It is generally recognized that i.n. vaccination leads to higher levels of sIgA antibodies at the mucosal surface with lower systemic IgG antibodies and cellular immune responses, while the opposite is true for i.m. vaccination (7, 38, 39). Remarkably, however, i.n. T4-CoV-2 vaccination induced higher systemic and mucosal immune responses (Fig. 2 and 3). This appears to be a distinctive feature of the T4 nanoparticle vaccine.

Indeed, the needle-free T4-CoV-2 vaccine induced robust mucosal IgG and sIgA responses. These anti-RBD or anti-Spike antibody titers were determined in bronchoalveolar lavage fluid (BALF) samples from vaccinated mice after the booster dose (Fig. 3). Intranasally administered vaccine elicited ~25-fold-higher IgG antibody levels (endpoint titers up to 12,500) in BALF compared to when animals were vaccinated i.m. (Fig. 3A and E), which also included both Th1-biased IgG2a and Th2-biased IgG1 subtype antibodies in a balanced manner (Fig. 3B, C, F, and G).

The sIgA antibodies play a critical role in protecting mucosal surfaces against pathogens by blocking their attachment and/or entry of viruses transmitted through the respiratory tract (40, 41). Thus, most significantly, high titers of mucosal sIgA antibodies were elicited by i.n. vaccination (endpoint titers up to 12,500) (Fig. 3D and H), in addition to high levels of systemic immune responses as described above (Fig. 2). In contrast, i.m. immunization failed to produce sIgA, which is not unexpected (Fig. 3D and H).

### Needle-free T4-CoV-2 vaccine provides complete protection and apparent sterilizing immunity against SARS-CoV-2 challenge.

**FIG 4:**
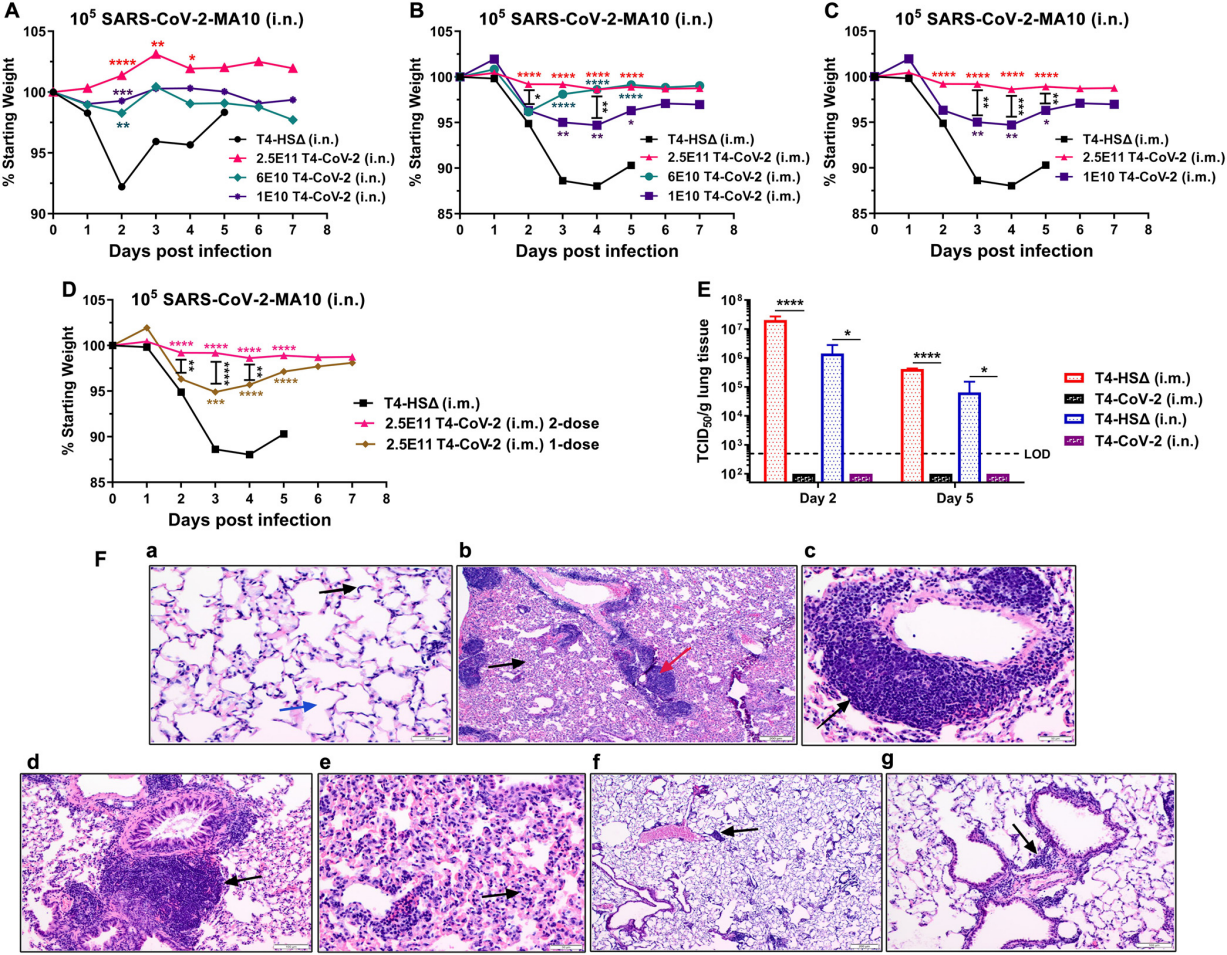
Needle-free T4-CoV-2 vaccination provided complete protection against SARS-CoV-2 challenge. (A) Percent starting body weights of i.n.-immunized mice at days after i.n. challenge with 10^5^ PFU of mouse-adapted SARS-CoV-2 MA10 strain. (B to D) Percent starting body weights of i.m.-immunized mice at days after i.n. challenge with 10^5^ PFU of SARS-CoV-2 MA10. Changes in the body weights for high, medium, and low doses of T4-CoV-2 vaccine are shown in panel B. Comparisons of body weights between high and low doses of T4-CoV-2 vaccine are shown in panel C. Comparisons of body weights between two-dose and single-dose regimens of T4-CoV-2 vaccine are shown in panel D. (E) Viral burden (TCID50/g lung tissue) in the lungs at 2 and 5 days after SARS-CoV-2 MA10 infection. T4-CoV-2 immunization was compared to the vector control in i.m. or i.n. groups. Dotted lines indicate the limit of detection (LOD) of the assay. (F) Histopathological analysis of lung tissues from the vector control and T4-CoV-2 i.n.-immunized and challenged mice. Representative photomicrographs from each group are shown. (a) Medium-power view of normal lung with delicate alveolar septa (black arrow) and distinct alveolar spaces (blue arrow) (200×). (b) Low-power view of lungs of the challenged control mice with prominent inflammatory infiltrates of bronchovascular bundles (red arrow), as well as interstitial involvement (black arrow; 40×). (c) Medium-power view of mononuclear inflammatory infiltrates around pulmonary vessel (black arrow; 200×) in challenged control mice. (d) Medium-power view of mononuclear cell infiltrate around bronchovascular bundle (black arrow; 100×) in challenged control mice. (e) Medium-power view of distal airways with evidence of interstitial inflammation in alveolar septa (black arrow) in challenged control mice. (f) Low-power view of lung with mild and patchy inflammatory infiltrate of bronchovascular bundles (black arrow) in challenged T4-CoV-2-immunized mice. Alveolar spaces and interstitium appear normal (40×). (g) Medium-power view of inflammatory infiltrate around bronchovascular bundle (black arrow; 100×) in challenged T4-CoV-2-vaccinated mice. For panels A to D, two-way ANOVA with Tukey’s *post hoc* test was used to compare multiple groups (*n* = 5 to 17; *n* = 17 for high-dose T4-CoV-2, *n* = 7 for medium- or low-dose T4-CoV-2, *n* = 10 for T4-HSΔ, and *n* = 5 for PBS). For panel E, one-way ANOVA with Tukey’s *post hoc* test (i.m.) and Mann-Whitney U test (i.n.) (*n* = 2 to 5) was performed. *, *P* < 0.05; **, *P* < 0.01; ***, *P* < 0.001; ****, *P* < 0.0001.

**(i) Animal challenge.** BALB/c mice were challenged with the mouse-adapted SARS-CoV-2 strain (MA10) (42) (Fig. 2B). As shown in Fig. 4A to D, the control animals that received the T4 vector exhibited a rapid weight loss soon after infection, with a maximum decrease on days 2 to 4 (Fig. 4A and B). On the other hand, mice immunized with the T4-CoV-2 vaccine by either of the two immunization routes showed modest to no weight loss over the course of 7 days after challenge.

More specifically, the weight loss curves among the high-, medium-, and low-dose i.n. vaccination groups were almost similar statistically. Compared to the T4 vector control, a much-reduced loss in body weights were noted on day 2 postinfection (p.i.) in all of the T4-CoV-2-vaccinated groups of mice, with subsequent minimal and statistically insignificant fluctuations in body weight changes until day 7 (Fig. 4A).

In i.m.-immunized groups, a similar comparison showed statistically significant differences on different days (Fig. 4B). Significantly less vaccine efficacy was apparent when the number of phage particles was reduced from 2.5 × 10^11^ to 1 × 10^10^ between days 3 to 5 p.i. (Fig. 4C). Similarly, significantly more weight loss was noticed in mice immunized i.m. with one dose of the T4-CoV-2 vaccine compared to mice receiving two doses on days 2 to 4 p.i. (Fig. 4D).

**(ii) Viral load.** To further assess protective efficacy in the lungs, the infectious virus load was determined by plaque assay on days 2 and 5 p.i., the peak period of viral burden in this model. As shown in Fig. 4E, no infectious SARS-CoV-2 virus could be detected in the lungs of mice immunized with the T4-CoV-2 vaccine (2.5 × 10^11^ phage particles) either i.m. or i.n. In fact, we observed quite the opposite; very high levels of virus, ~10^5^ to 10^7^ TCID_50_ [50% tissue culture infective dose(s)]/g, were present on day 2 in the control mice; this level decreased substantially on day 5 p.i., when the survived animals began to recover from infection. This indicates that the vaccine might be inducing sterilizing immunity, hence minimizing live virus shedding. This is consistent with the induction of strong mucosal immunity in the lungs of i.n.-vaccinated mice. Interestingly, the i.m.-vaccinated mice also showed no infectious SARS-CoV-2 virus in the lungs. The mechanism(s) leading to the induction of sterilizing immunity by both routes of vaccination warrants further investigation.

**(iii) Histopathology.** The lung tissues obtained from the control and immunized mice were subjected to hematoxylin-eosin (H&E) and MOVAT pentachrome staining for histopathological analysis. The analysis was performed based on three parameters: mononuclear inflammatory infiltrate around bronchovascular (BV) bundles, interstitial inflammation, and alveolar exudate/hemorrhage.

As shown in Fig. 4F, the uninfected normal lungs had delicate alveolar septa (black arrow) and distinct alveolar spaces (blue arrow) with no evidence of inflammation, hemorrhage, exudates, or transudates (Fig. 4Fa, 200×). On the other hand, prominent inflammatory infiltrates of bronchovascular bundles (red arrow), as well as interstitial involvement (black arrow), were noticed in the T4-vector control mice (i.n. immunized) during virus infection (Fig. 4Fb, 40×). More specifically, mononuclear inflammatory infiltrates were noticed around pulmonary vessel (black arrow, Fig. 4Fc, 200×) and bronchovascular bundle (black arrow, Fig. 4Fd, 100×). Distal airways with interstitial inflammation in alveolar septa (black arrow, Fig. 4Fe) were evident. In addition, alveolar hemorrhage was also observed in other areas of the lungs.

As for the T4-CoV-2 i.n.-immunized mice, only mild and patchy inflammatory infiltrate of bronchovascular bundles (black arrow; 40× and 100×, respectively) were noted after infection, and the alveolar spaces and interstitium appeared normal (Fig. 4Ff and g). Such minimal infiltrates in the lungs were also observed in SARS-CoV-2 mRNA and adenovirus vaccines (5, 15). Overall, the combined scores based on the above three parameters were 6.2 ± 1.3 for the T4 vector control and 4.4 ± 1.1 for the T4-CoV-2 vaccine i.n.-immunized animals (*P* = 0.01) when combined data on tissues after 2 and 5 days of challenge were analyzed.

Collectively, these data indicated that the T4-CoV-2 vaccine was effective in clearing the virus and potentially could block transmission of SARS-CoV-2.

### A Beta-variant needle-free T4-CoV-2 vaccine stimulates strong mucosal, humoral, and cellular immune responses in hACE2-transgenic mice.

**FIG 5:**
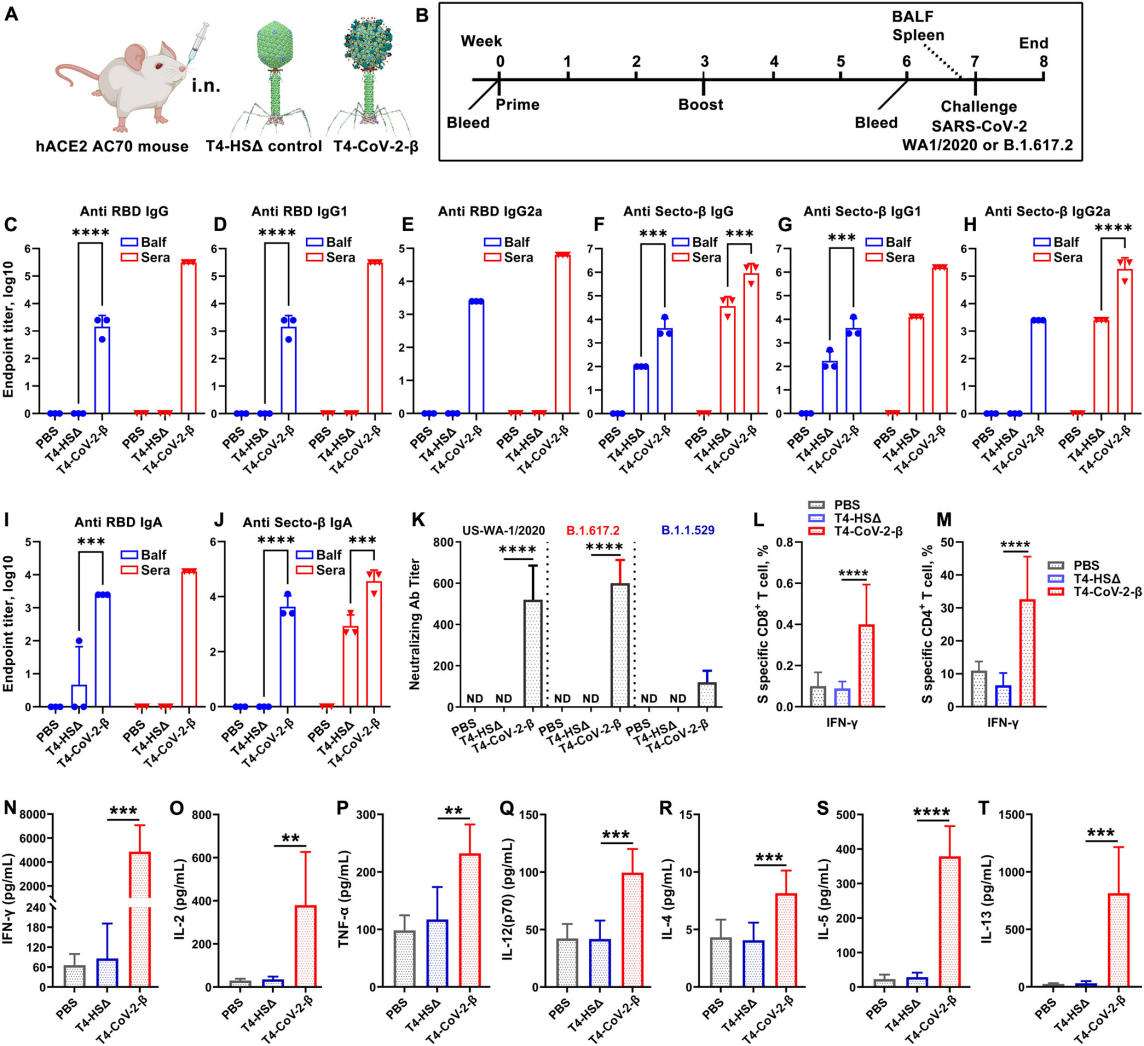
Intranasal T4-CoV-2-β vaccination stimulated robust mucosal and systemic humoral and cellular immune responses in hACE2-transgenic mice. (A) Schematic of i.n. mouse vaccination with T4-HSΔ control or T4-CoV-2-β vaccine. (B) Scheme for vaccination and challenge. (C to J) Antibody responses in sera (red) and BALF (blue) of immunized mice on day 21 after the boost. ELISA was applied to determine reciprocal endpoint antibody titers of anti-RBD IgG (C), anti-RBD IgG1 (D), anti-RBD IgG2a (E), anti-Secto-β IgG (F), anti-Secto-β IgG1 (G), anti-Secto-β IgG2a (H), anti-RBD IgA (I), and anti-Secto-β IgA (J). (K) Neutralizing antibody titers in sera were determined by a Vero E6 cell cytopathic assay using WA-1/2020, B.1.617.2 (Delta), and B.1.1.529 (Omicron) strains. (L and M) Cellular immune responses after stimulation with Secto-β protein. The percentages of IFN-γ^+^ CD8^+^ (L) and IFN-γ^+^ CD4^+^ (M) cells were plotted. (N to T) Splenocyte cytokine responses to Secto-β protein stimulation in immunized hACE2-transgenic mice. Representative Th1 (N to Q) and Th2 (R to T) cytokines are shown. For panels C to M, two-way (C to J, L to M) or one-way ANOVA (K) was performed with a Tukey *post hoc* test. For panels N to T, a nonparametric Student *t* test was performed. The data are from three pooled independent experiments (*n* = 15 for T4-HSΔ and PBS sera analysis, *n* = 21 for T4-CoV-2-β sera analysis, and *n* = 5 for BALF analysis). The data are representative of two (K) or five (L to T) biological replicates. **, *P* < 0.01; ***, *P* < 0.001; ****, *P* < 0.0001.

To determine whether the robust and diverse immune responses elicited by the T4-CoV-2 vaccine, especially the mucosal responses, could be recapitulated in highly susceptible hACE2 knock-in mice, we conducted an independent study. In addition, we constructed a Beta-variant Spike trimer (Secto-β) (without any affinity tags) for vaccination since this was a dominant strain at the time of the study causing a major second wave in South Africa and across the globe (43). Secto-β contained four critical mutations (K417N, E484K, N501Y, and D614G) that conferred enhanced transmissibility and lethality and also partial escape from vaccine-induced immunity (44) (see Fig. S3A). The Secto-β variant trimer conjugated to T4 capsid as efficiently as the WT S-trimer through the Spytag-SpyCatcher system (20) (see Fig. S3B). In addition, the T4-CoV-2-β vaccine also contained ~100 copies of NP protein packaged inside the capsid (see Fig. S3C). Five-week-old hACE2 AC70 mice were i.n. immunized with this vaccine using the same prime-boost regimen (Fig. 5A and B) at a high dose (~2.5 × 10^11^ phage particles decorated with 20 μg of variant Secto-β).

10.1128/mbio.01822-22.3 FIG S3 Characterization of the Secto-β trimer, copy number of NP in T4-CoV-2-β vaccine, and anti-NP antibody responses. (A) Schematics of Secto and Secto-β cassette for expression in ExpiCHO cells. The Secto-β was constructed by incorporating K417N, E484K, and N501Y mutations in the receptor-binding domain (RBD) and D614G mutation in the S2 region of the trimer. These mutations are the core mutations of Beta variant which are responsible for the immune escape in vaccinated people. (B) *In vitro* display of Secto and Secto-β trimers on T4-SpyCatcher phage at increasing ratios of S protein molecules to Soc binding sites (0:1 to 2:1). S trimer and T4-SpyCatcher phage were incubated at 4°C for 1 h, followed by centrifugation to remove the unbound material. After two washes, the pellet was resuspended in 1 × PBS buffer (pH 7.4), and SDS-PAGE was performed. The positions of Soc-SpyCatcher (red arrowhead) and S-protein bands (red line) are indicated. (C) Quantification of the copy number of NP protein molecules packaged in T4-CoV-2 vaccine by Western blotting with commercial NP standard (Sino Biological). (D) Anti-NP antibody responses in sera (red) and BALF (blue) of immunized mice at day 21 after boosting. ELISAs were performed to determine reciprocal endpoint antibody titers of anti-NP IgG. PBS and T4-HSΔ were used as naive and vector controls, respectively. Data represent the means ± the SEM from three pooled independent experiments (*n* = 15 for sera analysis and *n* = 5 for BALF analysis). *P* values were calculated using two-way ANOVA with the Tukey post *hoc* test to compare multiple groups (**, *P* < 0.01). Download FIG S3, TIF file, 2.8 MB. Copyright © 2022 Zhu et al. 2022 Zhu et al. https://creativecommons.org/licenses/by/4.0/ This content is distributed under the terms of the Creative Commons Attribution 4.0 International license.

**(i) Humoral immune responses.** Similar to the binding antibody titers in BALB/c mice (Fig. 2 and 3), i.n. immunization with T4-CoV-2-β induced high levels of Spike- and RBD-specific IgG and IgA in the sera of hACE2-transgenic mice (Fig. 5C to J), suggesting a strong systemic humoral immune response. In addition, moderate NP-specific IgG antibodies were also elicited in the T4-CoV-2-β-immunized mice (see Fig. S3D). Furthermore, high levels of Spike- and RBD-specific IgG and sIgA antibodies were also present in BALF of T4-CoV-2-β-vaccinated mice, indicating an equally robust mucosal immune response (Fig. 5C to J; see also Fig. S4A to D). There was no significant difference in binding antibody titers between Secto and Secto-β as the coating antigen (see Fig. S4E and F), probably because they share a large number of the same epitopes. Collectively, consistent with our findings in BALB/c mice, T4-CoV-2-β i.n. vaccination stimulated strong mucosal and systemic humoral immune responses in hACE2-transgenic mice.

10.1128/mbio.01822-22.4 FIG S4 Secto- and Secto-β-trimer binding antibody titers in T4-CoV-2-β i.n.-immunized hACE2-transgenic mice. (A to D) Antibody responses in sera (red) and BALF (blue) of immunized mice at day 21 after boosting. ELISAs were performed to determine reciprocal endpoint antibody titers of anti-Secto IgG (A), anti-Secto IgG1 (B), anti-Secto IgG2a (C), and anti-Secto IgA (D). The data are from three pooled independent experiments (*n* = 15 for sera analysis and *n* = 5 for BALF analysis). *P* values were calculated using two-way ANOVA with a Tukey *post hoc* test to compare multiple groups (*, *P* < 0.05; **, *P* < 0.01; ***, *P* < 0.001). (E and F) Comparison between anti-Secto and anti-Secto-β antibody responses (IgG, IgG1, IgG2a, and IgA) in sera (E) and BALF (F). ns, no significance. Download FIG S4, TIF file, 2.0 MB. Copyright © 2022 Zhu et al. 2022 Zhu et al. https://creativecommons.org/licenses/by/4.0/ This content is distributed under the terms of the Creative Commons Attribution 4.0 International license.

Importantly, consistent with the broad-spectrum neutralizing activities in BALB/c mice (Fig. 1K), T4-CoV-2-β vaccine elicited virus neutralizing activities comparable to WA-1/2020 and its Delta (B.1.617.2) VOC in hACE2-transgenic mice, while no detectable neutralizing activities were detected in PBS or T4 vector control groups (Fig. 5K). In addition, the Omicron (BA.1) variant emerged in late November of 2021 (near the end of this study) and has the largest number (>30) of mutations within the Spike protein described to date. These mutations substantially jeopardized the efficacy of existing COVID-19 vaccines (45, 46), resulting in a major spike in breakthrough infections. Our T4-CoV-2-β-vaccinated sera neutralized the Omicron variant (B.1.1.529) but the titers were 6-fold lower compared to the WA-1/2020 strain (Fig. 5K), which was also reported for mRNA vaccine against Omicron (8-fold reduction) (45). Interestingly, neutralization of Omicron was comparable to that of WA-1/2020 in BALF (see Fig. S5A), although the BALF titer appeared lower than that of sera, largely due to dilution of the lung lining fluid. The effect of T4-CoV-2 vaccine on Omicron transmission will be further investigated in our future study.

10.1128/mbio.01822-22.5 FIG S5 Virus neutralization activity in BALF and T cell immune responses in hACE2-transgenic mice. (A) Neutralizing antibody titers in BALF were determined by Vero E6 cell cytopathic assay using ancestral SARS-CoV-2 US-WA-1/2020 and B.1.1.529 (Omicron) strains. Data represent the means ± the standard deviations from two pooled replicate experiments. (B and C) Analysis of CD8^+^ (B) and CD4^+^ (C) T cell immune response in stimulation with Secto-β protein. Cells were then stained with the T cell surface markers CD3, CD4, and CD8, followed by intracellular IFN-γ, TNF-α, and IL-17A staining. The percentages of IFN-γ^+^, TNF-α^+^, or IL-17A^+^ cells in CD4^+^ or CD8^+^ cells were plotted. *P* values were calculated using two-way ANOVA with a Tukey *post hoc* test to compare multiple groups (*, *P* < 0.05; **, *P* < 0.01; ****, *P* < 0.0001). Data represent the means ± the standard deviations of five biological replicates. Download FIG S5, TIF file, 0.9 MB. Copyright © 2022 Zhu et al. 2022 Zhu et al. https://creativecommons.org/licenses/by/4.0/ This content is distributed under the terms of the Creative Commons Attribution 4.0 International license.

**(ii) Cell-mediated immune responses.** As shown in Fig. 5L and M, restimulation of splenocytes *ex vivo* with S protein showed a similar pattern of CD8^+^ and CD4^+^ T cell activation in hACE2 mice, as with the conventional BALB/c mice (Fig. 2L and M). The percentages of CD8^+^ and CD4^+^ T cells positive for IFN-γ were substantially elevated in T4-CoV-2-β-immunized mice compared to both PBS and T4 vector control groups (*P* < 0.0001) (Fig. 5L and M). Interestingly, a much higher percentage of IFN-γ-positive CD4^+^ T cells was observed in hACE2 mice than in conventional BALB/c mice, while the percentages of TNF-α- or IL-17A-positive T cells were similar (see Fig. S5B and C). T4-CoV-2-β i.n. immunization developed robust Spike-specific CD8^+^ and CD4^+^ T cell responses in hACE2-transgenic mice.

Similarly, both Th1 cytokines (IFN-γ, IL-2, TNF-α, and IL-12-p70) (Fig. 5N to Q) and Th2 cytokines (IL-4, IL-5, and IL-13) (Fig. 5R to T) were induced in T4-CoV-2-β-immunized mice compared to the controls when splenocytes were retreated with the Secto-β trimer. Significantly, the very prominent Th1 cytokines IFN-γ (4,848 pg/mL) and IL-2 (380 pg/mL) were produced, indicating Th1-biased cellular immune responses induced by the i.n. T4-CoV-2-β vaccine.

### Needle-free T4-CoV-2-Beta vaccine provides complete protection and apparent sterilizing immunity against lethal infection by both the original SARS-CoV-2 and the Delta VOC in hACE2-transgenic mice.

**FIG 6:**
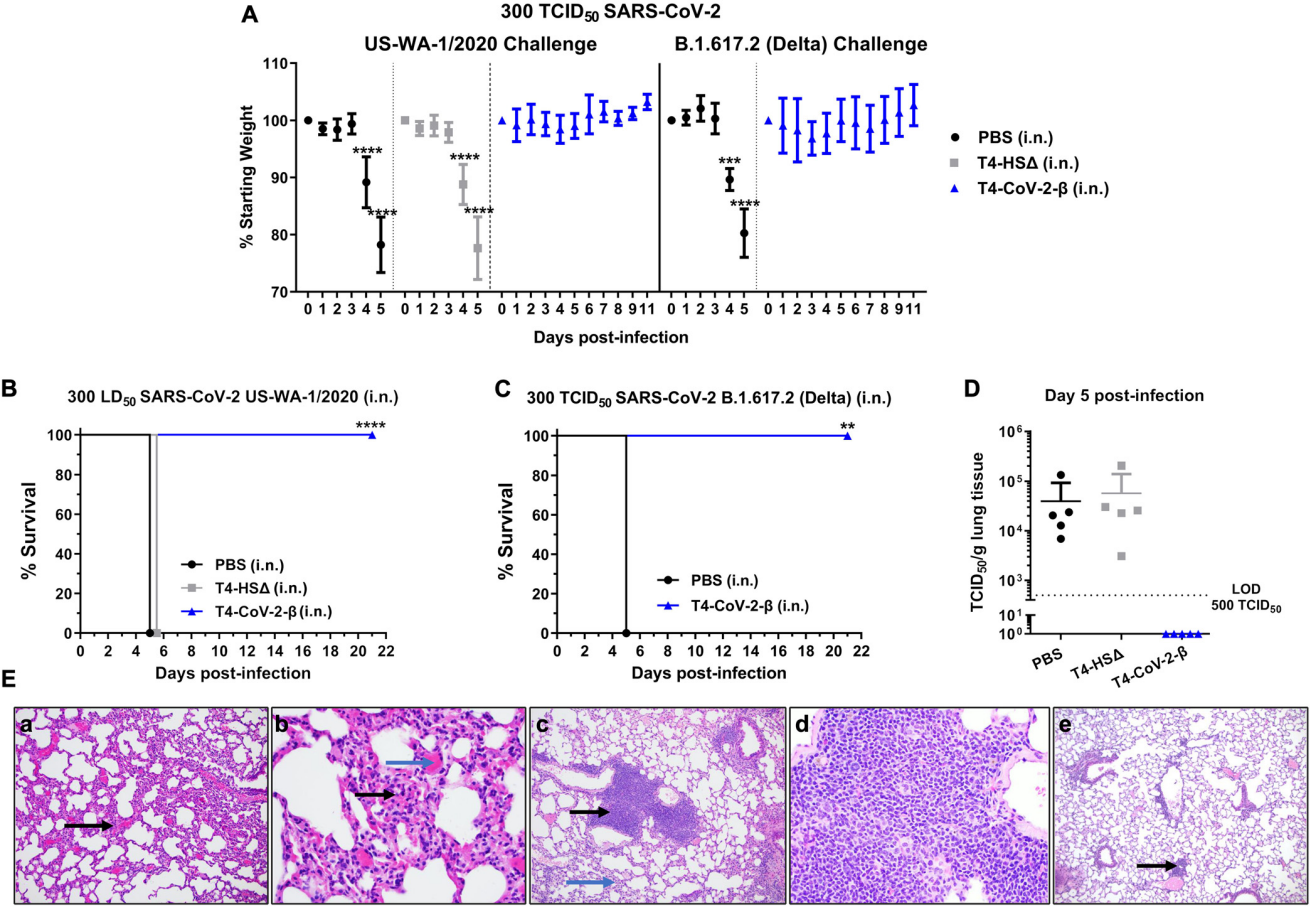
Needle-free T4-CoV-2-β vaccine provided complete protection against lethal infection by ancestral SARS-CoV-2 strain, as well as its Delta variant, in hACE2-transgenic mice. (A) Percent starting body weights of immunized mice on various at days after i.n. challenge with 300 TCID_50_ of WA-1/2020 strain or its Delta (B.1.617.2) variant. (B and C) Survival rates of hACE2-transgenic mice immunized with T4-CoV-2-β or T4-HSΔ vector control against WA-1/2020 strain (B) or its Delta variant (B.1.617.2) (C). (D) Viral burden (TCID_50_/g lung tissue) in the lung at 5 days after WA-1/2020 infection. Dotted lines indicate the limit of detection (LOD) of the assays. (E) Lung tissues obtained from the control (a and b) and T4-CoV-2-β (c to e)-immunized mice (i.n.) challenged with the WA-1/2020 strain were subjected to H&E and MOVAT staining for histopathological analyses, and representative photomicrographs from each group are shown. At day 5 p.i., significant interstitial inflammation in the alveolar septa was evident (black arrow, panel a), and widening of the interstitium with mononuclear inflammatory infiltrates (black arrow) and septal capillary congestion was clearly visible (blue arrow, panel b) in the control group. T4-CoV-2-β i.n.-vaccinated and challenged animals had mild interstitial inflammation (blue arrows, panel c) and bronchovascular inflammatory infiltrates (black arrows, panel c) were clearly visible. At day 30 p.i., there was no evidence of interstitial pneumonitis and only a mild bronchovascular inflammation (black arrow, panel e) in T4-CoV-2-β-vaccinated and challenged mice. For panel A, a multiple Student *t* test using the Holm-Sidak method to correct for multiple comparisons (*n* = 3 to 10) was performed. For panels B and C, Kaplan-Meier analysis with a log-rank (Mantel-Cox) test (*n* = 3 to 10) was performed. **, *P* < 0.01; ***, *P* < 0.001; ****, *P* < 0.0001.

**(i) Animal challenge and viral load.** Mice were challenged i.n. with either WA-1/2020 strain or its Delta (B.1.617.2) variant. The highly contagious B1.617.2 shows increased transmissibility compared to the ancestral strain, and studies suggested a high risk of hospitalization compared to the original strain (47). As shown in Fig. 6A, irrespective of the challenge strains, all control animals rapidly lost weight (Fig. 6A) and succumbed to infection (Fig. 6B and C) on days 4 to 5 postchallenge. In contrast, all the T4-CoV-2-β-immunized mice only had minimal to no weight loss, with a 100% survival rate over 21 days after the challenge. Furthermore, a high viral load in the lungs was observed in all control animals on day 5 after WA-1/2020 strain infection, whereas no live virus was detected in the lungs of T4-CoV-2-β-vaccinated mice (Fig. 6D).

**(ii) Histopathology.** As seen in Fig. 6E, hACE2-transgenic mice treated with PBS and then challenged with WA-1/2020 strain showed significant interstitial inflammation in alveolar septa (black arrow, Fig. 6Ea, 100×) and alveolar hemorrhage. However, there was no evidence of bronchovascular inflammatory infiltrates on day 5 p.i. At 200× magnification, widening of interstitium with mononuclear inflammatory infiltrates (black arrow) and septal capillary congestion was clearly visible (blue arrow, Fig. 6Eb) in PBS-treated and challenged mice.

Based on interstitial inflammation, animals receiving PBS or immunized with T4 vector and then challenged had similar scores of 40 ± 7.1 (PBS group) and 46 ± 18 (T4 vector control group) on day 5 p.i., and the data were not significantly different (*P* = 0.5, Student *t* test). Further, upon comparing unvaccinated animals (PBS + vector control groups together) to animals receiving the T4-CoV-2-β vaccine, we found that interstitial inflammation was significantly less in immunized mice (*P* = 0.007, Mann-Whitney rank sum test, the results were expressed as median, 25%, and 75% with values of 40, 30, and 52.5 for PBS and T4 vector control immunized and challenged mice compared to 20, 20, and 30 for the T4-CoV-2-β vaccinated and challenged animals) on day 5 p.i.

Although T4-CoV-2-β i.n.-vaccinated and -challenged animals had mild interstitial inflammation (blue arrows, Fig. 6Ec), bronchovascular inflammatory infiltrates (black arrows, Fig. 6Ec, 100×) were clearly visible; this was not noted in unvaccinated and challenged mice. The bronchovascular infiltrates were mainly composed of lymphocytes and scattered macrophages (200×, Fig. 6Ed). Statistically, mice vaccinated with the T4 vector or T4-CoV-2-β and then challenged had a higher level of bronchovascular infiltrates than PBS treated and infected animals, indicating that T4 phage could increase bronchovascular infiltrates in hACE2-transgenic mice. Importantly, at day 30 p.i., there was no evidence of interstitial pneumonitis and only a mild bronchovascular inflammation (black arrow, Fig. 6Ee, 40×) in T4-CoV-2-β vaccinated and challenged mice. These data indicated almost complete recovery of animals from bronchovascular infiltrates.

Overall, our data indicated immunological responses induced by the vaccine cleared the infection with 100% survival of the animals. T4 vector, like any other vectors, is expected to activate some nonspecific and nondamaging immune responses in the host that subside as the vaccine clears from the host.

### The T4-CoV-2 vaccine is stable at ambient temperature.

The current mRNA vaccines require subfreezing temperatures, while the adenovirus-based vaccines require cold temperatures, for storage and distribution. Bacteriophage T4 being a resident of the gut has evolved a stable capsid structure to survive in a hostile environment. Indeed, the T4 phage is stable at extremes of pH and at ambient temperature, properties that are particularly suitable for storage and extending the life of a vaccine (48).

**FIG 7:**
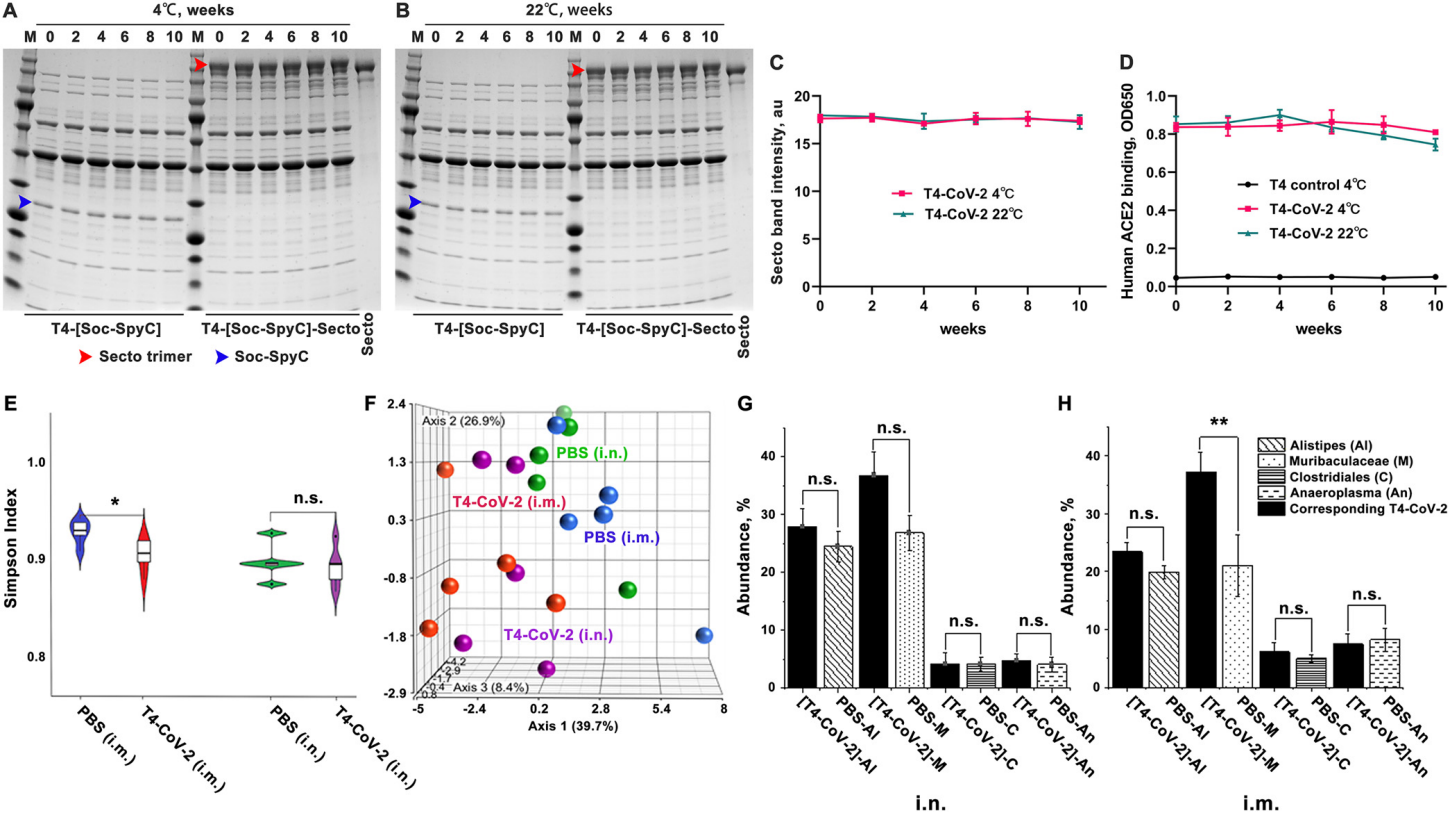
T4-CoV-2 vaccine is stable at ambient temperature and does not influence the microbiome community in mice. (A and B) Stability of T4-CoV-2 and T4-(Soc-SpyC) phages for 10 weeks at 4°C (A) or 22°C (B). Samples were taken every 2 weeks and analyzed for stability by SDS-PAGE. The blue and red arrowheads indicate the bands of Soc-SpyCatcher and covalently conjugated Secto protein, respectively. (C) Quantification of the displayed Secto band in T4-CoV-2 vaccine stored at 4 or 22°C. (D) Comparison of binding efficiency of T4-CoV-2 phage to hACE2 receptor after storage at 4 or 22°C. (E) The correlated distribution of the Simpson diversity index of microbiomes from PBS control and T4-CoV-2-vaccinated groups when immunization occurred by the i.n. or i.m. route. The measure of diversity included number and relative species abundance. (F) Summary of individual Euclidian distance as a 3D resemblance matrix of microbial species in the tested groups. (G and H) Specific effect of the vaccination of T4-CoV-2 and PBS control on the bacterial genera of the microbiome. The abundances of gut microbiota following i.n. (G) or i.m. (H) administration of the T4-CoV-2 vaccine and the PBS control are shown.

To determine the stability of the T4-CoV-2-β, the vaccine preparations in PBS were stored at 4°C and room temperature (22°C), and samples were taken at various time points and analyzed for stability and receptor-binding functionality. Stability was assessed by any reduction in the amount of intact Spike protein associated with phage (due to dissociation) and/or the appearance of any degraded protein fragments (due to nonspecific proteolysis), whereas receptor-binding functionality was assessed by the ability of the displayed S-trimers to bind to hACE2 receptor. The data showed (Fig. 7A to D) that the T4-CoV-2-β vaccine, by any of these criteria, was completely stable in terms of physical structure and ACE2 receptor-binding functionality for at least 10 weeks of storage at 4°C or at 22°C. Furthermore, the backbone phage displaying the SpyCatcher domain as part of the hard-wired recombinant phage, i.e., prior to conjugation with S-trimer, also remained completely stable structurally.

### T4-CoV-2 vaccination does not influence the microbiome community.

Finally, we sought to determine whether T4-CoV-2 vaccination impacted the microbiome community. DNA was extracted from the fecal matter of individual mice (*n* = 5/group), sequenced for 16S rRNA gene, and analyzed.

**(i) Violin plot.** The violin plot in Fig. 7E shows the correlated distribution of the Simpson diversity index of microbiomes in the test groups. The measure of diversity included number and relative species abundance. As noted, i.n. administration did not alter the Simpson diversity of the microbial species recovered from the PBS control versus the T4-CoV-2 vaccine groups of mice, in contrast to i.m. vaccination. These results indicated that i.n. vaccination did not significantly affect the number and relative abundance of the gut microbiota.

**(ii) Principal coordinate analysis.** Figure 7F summarized individual Euclidian distance as a three-dimensional (3D) resemblance matrix of microbial species. The data indicated that the relative distances based on the number between species during both routes of immunization were similar, but there was a significant difference in species diversity when i.m. immunization occurred (PBS control versus T4-CoV-2 groups). However, this was not the case for i.n. immunization since there was a lack of significant differences among species.

**(iii) Specific effect on the bacterial genera of the microbiome.** Figure 7G and H show abundance of the gut microbiota. The Tukey mean comparison method between the T4-CoV-2 and PBS groups for the top four genera (*Alistipes*, *Muribaculaceae*, *Clostridiales*, and *Anaeroplasma*) indicated no significant differences in the gut microbiota, even though there were few differences in numbers (e.g., for *Alistipes* and *Muribaculaceae*) when vaccine was administered i.n. (Fig. 7G). However, a significant difference in the *Muribaculaceae* genus was noted when T4-CoV-2 vaccine was delivered i.m. (Fig. 7H). These same differences were observed among the *Bacteroidetes* phylum indicating that i.m. administration of the T4-CoV-2 vaccine had a more significant impact on gut microbiota. These trends were also reflective upstream of the hierarchy from families to the phylum of the recovered gut microbiota. Increasing evidence shows that the gut microbiota plays an important role in regulating immune responses to various vaccines (49). Postvaccination microbiota perturbation was previously reported during early microbial and immunological maturation stages in humans (50). Similarly, Chen et al. identified postvaccination dysbiosis as a significant problem in developing cellular immunity in COVID-19 vaccines, which can be corrected by introducing prebiotics and probiotics oral supplements after vaccination (51). Notably, the T4-based COVID-19 vaccine administered by the i.n. route seemed to circumvent this effect on the microbiota. In the future, the effect of vaccination on the nose/lung microbiota will also need to be examined.

## DISCUSSION

A next-generation COVID-19 vaccine that would elicit local mucosal responses in addition to strong systemic immunity is most desired to control SARS-CoV-2 infections and, in general, any mucosally transmitted infection (3, 9, 11, 13). This is particularly relevant at this stage of the COVID-19 pandemic, in view of the current evolutionary trajectory of the virus selecting highly transmissible variants such as Omicron BA.1 and BA.2.

The sticky mucous layers in the nasal epithelia present barriers to pathogens and possibly interfere with the ability of vaccines to access and activate the mucosal immune system. This may account for poor immunogenicity of most injectable vaccines when administered i.n. (7, 10). At present, of ~195 COVID-19 vaccine candidates in clinical trials, only 14 are i.n. vaccines. Most of them are based on engineered live viruses that can efficiently infect human cells and intracellularly express Spike or RBD antigens from the delivered genes. These include human or chimpanzee adenoviruses (15, 17, 41, 52–55), live-attenuated influenza virus (56, 57), live-attenuated Newcastle Disease virus (58, 59), and lentivirus (12). However, these eukaryotic viral vaccines still pose a safety concern, preexisting immune responses, and a risk, albeit very low, of reversion.

Our studies established a prokaryotic, noninfectious, bacteriophage T4 mucosal vaccine delivery platform that can be engineered to generate stable, needle- and adjuvant-free, multicomponent vaccines against COVID-19 or any emerging and pandemic pathogen. The presence of ~17-nm-long Hoc fibers on the T4 capsid surface that could interact with mucin glycoproteins and S-trimers binding to ACE2 receptors provides distinct advantages for i.n. delivery and presentation to host’s mucosal immune system. Indeed, a series of data sets demonstrate that the T4-CoV-2 nanoparticle vaccine containing arrays of ~100 copies of S-trimers on T4 capsid exterior and ~100 copies of NP packaged in its interior when administered to mice i.n. stimulated all arms of the immune system, including strong mucosal immunity that injectable vaccines do not induce.

The immune responses stimulated by the T4 based COVID-19 vaccine were broad and included the following: Th1 and Th2 derived IgG and IgA antibodies in sera, virus-neutralizing antibodies, CD4^+^ helper and effector T cells and CD8^+^ killer T cells, Th1-biased cytokines, and mucosal IgG and sIgA antibodies in BALF. Surprisingly, while most of these immune responses were triggered by both i.n. and i.m. routes of vaccine administration, the stimulation was considerably stronger by i.n. immunization. Remarkably, however, the mucosal sIgA in BALF was stimulated only by i.n. vaccination. The sIgA is supposed to be effective at the entry point by interfering with virus acquisition and at the exit point by clearing the invaded pathogen (Fig. 1) (14, 60). This pattern of broad responses was consistently observed for both the WT and the Beta-variant S-trimers and in both conventional BALB/c and hACE2-transgenic mice. The evidence suggesting that strong vaccine-induced mucosal and systemic immunity is a prominent feature of the needle-free bacteriophage T4 nanoparticle vaccine is thus compelling, and this could be further exploited for designing vaccines against other respiratory infections (61).

Strikingly, the T4-CoV-2 vaccine induced similar levels of serum virus neutralizing antibody titers against the ancestral WA-1/2020 strain and its two VOCs (B.1.135 Beta and B.1.617.2 Delta), which can significantly escape immune responses by the existing mRNA or adenovirus vaccines (62, 63). Consistently, our vaccine protected mice from challenge by both the WA-1/2020 strain and its Delta (B.1.617.2) variant, which is considered thus far the most lethal strain. In addition, the T4-CoV-2 vaccine also induced significant but somewhat diminished neutralizing antibody titers against the Omicron variant, which has the greatest number of mutations and immune-escaping capacity reported to date (45). Importantly, similar levels of neutralizing antibody titers were measured in BALF against both WA-1/2020 isolate and its Omicron variant. It is intriguing why the neutralization activity induced by T4-CoV-2 vaccination is so broad. The reasons for this finding require further investigation, especially if it relates to the presence of high levels of IgA in the serum and sIgA in BALF, which are reported to be more potent than IgG in neutralizing SARS-CoV-2 virus (14, 64).

Notably, the T4-CoV-2 nanoparticle vaccine is also a potent inducer of cellular immunity. Our studies demonstrated that both routes of immunization (i.n. and i.m.) induced the enhanced release of proinflammatory/anti-inflammatory and Th1/Th2 cytokines in BALB/c and hACE2-transgenic mice. Interestingly, i.n. immunization induced greater cellular responses, especially Th1, compared to i.m. vaccination. Th1 cells and cytotoxic T lymphocytes are primarily responsible for host defense against viral infections, and the role of Th2 cells in recruiting different types of innate immune cells to kill invading pathogens is also well documented (65). A Th1 cell-biased response or balanced Th1/Th2 cell response has also been reported by others upon immunization of mice, hamsters, and macaques with effective COVID-19 vaccines (4, 5, 13, 15, 45, 66). Therefore, a combination of producing neutralizing antibodies and activation of antigen-specific T cells may act in concert to control SARS-CoV-2 infection in our mouse models.

In addition, we also observed Th17 immune responses elicited by T4-CoV-2 vaccine. Th17 cells are being recognized as an important T helper subset for immune-mediated protection, and unbalanced Th17 responses are implicated in the pathogenesis of several autoimmune and allergic disorders (67). Involvement of IL-17 in priming enhanced chemokine and granulocyte colony-stimulating factor production in the lung during bacterial pneumonia and its ability to promote antimicrobial responses against pathogens of viral, bacterial, parasitic, and fungal etiology have been reported (68, 69). For example, mucosal delivery of M. tuberculosis subunit vaccine has been shown to provide IL-17 dependent protection of mice against pulmonary tuberculosis compared to when the vaccine was delivered by the parenteral route (70). Since the T4-CoV-2 vaccine provided complete protection to mice with much-reduced histopathological lesions, our data support the notion that a delicate balance of Th1/Th2/Th17 and mucosal immune responses was critical in developing effective COVID-19 vaccines.

The T4-CoV-2 vaccine is a safe and stable vaccine. A noninfectious phage T4-CoV-2 vaccine with no tropism to human cells and no use of adjuvants or chemical stimulants represent significant advantages. Two recent phage-COVID vaccine studies using filamentous AAVP (71) and Bxb1 phage (24) also demonstrated the advantages of phage vaccine system, including the low cost of production, the self-adjuvanted nature, and the strong safety profile. In fact, our previous studies showed that adding adjuvants such as alum or liposomes did not further enhance the levels of immune responses (20, 72). Microbiome analyses showed no significant changes in microbiome diversity in mice vaccinated with the T4-CoV-2 vaccine. Rapid clearance of the T4-CoV-2 vaccine when administered by the i.n. route over the i.m. route from mice after stimulation of resident memory T cells and antibodies by B cells could be why the microbiota was not impacted after i.n. immunization. However, further studies are needed to address this point. Human clinical trials and hundreds of T4 phage vaccine immunizations over the years involving mice, rats, rabbits, and macaque animal models and diverse antigens such as anthrax, plague, and HIV did not identify any significant side effects (72–75). Furthermore, the T4 phage is one of the most stable virus scaffolds known (48), and our stability studies showed that the T4-CoV-2 vaccine was completely stable at an ambient temperature for at least 10 weeks. Therefore, the T4 vaccine that requires no cold chain provides an excellent alternative for global distribution and vaccination of still-unvaccinated populations across the world. However, the stability of the T4 vaccine with respect to immunogenicity and at a higher temperature (e.g., 40°C, which is the ambient temperature in many parts of the world) and a safety concern such as the potential retrograde transportation of the vaccine nanoparticle to the brain, as reported with the i.n. vaccination of influenza virosomes along with the E. coli mutated heat-labile toxin mucosal adjuvant (76, 77), require further investigations.

In addition, the T4-CoV-2 vaccine is a strong candidate as an effective booster vaccine. Before the pandemic ends, an additional booster will likely be needed to protect the global population from emerging variants. None of the licensed vaccines used worldwide are needle-free or generate significant mucosal responses, which are critically important for minimizing person-to-person transmission. The T4-CoV-2 vaccine that can boost not only the antibody and T cell immune responses but also induce strong mucosal immunity would be the most beneficial one. Furthermore, more than a billion vaccinations across the globe received the adenovirus-based vaccines, which also stimulate strong antivector responses. This preexisting immunity, particularly the adenovirus capsid neutralizing antibodies, limits the effectiveness of another booster dose using the same vaccine, especially in the elderly, because vaccine delivery requires efficient infection of human cells which would be compromised by immune clearance (78). Furthermore, due to safety concerns, strict limitations were recently imposed on the administration of an adenovirus-derived COVID vaccine (79). Since T4 is noninfectious and there is no significant preexisting immunity in humans for T4 (80), the T4-CoV-2 vaccine would be an excellent alternative to boost more than a billion people who already received the adenoviral vaccines.

In conclusion, we have established a bacteriophage T4-based, protein vaccine platform, complementing the current mRNA and DNA vaccine platforms but with certain advantages in terms of route of administration, engineerability, breadth of immune responses, mucosal immunity, and vaccine stability. In particular, broad virus neutralization activity, both systemic and mucosal, T cell immunity, complete protection, and apparent sterilizing immunity, all induced by the same vaccine mean that the T4-CoV-2 vaccine might be able to block viral entry (host’s viral acquisition) and viral exit (host’s viral shedding), minimizing person-to-person viral transmission. However, additional studies in animal models (hamsters and macaques), phase 1 human clinical trials, and good manufacturing processes are needed to translate the vaccine into mass production and global distribution. These efforts are under way and crucial since more than 10-billion doses of the vaccines are needed across the globe, particularly in middle- to low-income countries, where the affordability of the current vaccines is a big concern.

## MATERIALS AND METHODS

### T4 bacteriophages and SARS-CoV-2 recombinant strains.

The T4-CoV-2 vaccine is a recombinant T4 phage displaying 100 copies of prefusion-stabilized SARS-CoV-2 Spike protein ectodomain trimers (S-trimers) on the surface of 120 × 86-nm phage capsid. It also harbors SARS-CoV-2 nucleocapsid protein (NP) packaged in its core and a 12-amino-acid peptide of the putative external domain of E protein (Ee) on the capsid surface. The S-trimers were displayed through interaction with the small outer capsid protein (Soc) which is attached to EXPiCHO-expressed S-trimers via SpyCatcher-SpyTag conjugation. The Ee peptide was attached through fusion to the highly antigenic outer capsid protein (Hoc). The NP, Ee, and SpyCatcher were hard-wired into the T4 genome by CRISPR engineering and incorporated into the phage nanoparticle structure during phage infection to make vaccine production easy. The T4 phage without carrying the SARS-CoV-2 components was used as a vector control.

Mouse adapted SARS-CoV-2 MA10 strain is a gift from R. Baric, University of North Carolina, Chapel Hill, NC. The first COVID-19 patient isolate SARS-CoV-2 US-WA-1/2020, its Beta (B.1.351), Delta (B.1.617.2), and Omicron (B.1.1.529) VOCs were obtained through the Centers for Disease Control and Prevention and are available at the Galveston National Laboratory, University of Texas Medical Branch (UTMB).

### T4 bacteriophage production, purification, display, and stability evaluation.

Bacteriophages T4-NP-Ee-(Soc-SpyCatcher), T4-NP-(Soc-SpyCatcher), and T4-HSΔ were produced in Escherichia coli strain B40 and purified by two rounds of CsCl gradient centrifugation as described previously (81, 82). The purified phages were passed through a 0.22-μm-pore-size filter to remove any minor bacterial contaminants. *In vitro* display of Secto or Secto-β trimers on T4-NP-Ee-(Soc-SpyCatcher) and T4-NP-(Soc-SpyCatcher) phages, respectively, was assessed by cosedimentation as described previously (20). The Secto displayed T4-NP-Ee-(Soc-SpyCatcher) phage is referred to as T4-CoV-2 vaccine and the Secto-β displayed T4-NP-(Soc-SpyCatcher) phage is referred to as T4-CoV-2-β vaccine. The phage concentration and copy numbers of displayed antigens were quantified by using 4 to 20% SDS-PAGE. The copy numbers of displayed antigens per capsid were calculated using gp23 (major capsid protein; 930 copies) or gp18 (major tail sheath protein; 138 copies) as internal controls and the S-trimer protein standard. The copies of the phage-packaged NP protein were quantified by Western blotting with the commercial rabbit anti-NP antibody (Sino Biological) and NP protein standard (Thermo Fisher Scientific) as previously described (20).

For physical stability evaluation, the T4-CoV-2 vaccine phage (T4-[Soc-SpyC]-Secto) and the T4 backbone phage (T4-[Soc-SpyC]) were flash-frozen at −70°C at the time zero as 100% controls. Two sets of the same phages were stored at 4 or 22°C, and samples were taken at 2-week intervals for 10 weeks and then flash-frozen at −70°C. All the samples were thawed and analyzed together for stability by SDS-PAGE. After Coomassie blue R-250 (Bio-Rad) staining and destaining, the displayed S-trimer protein bands on SDS-PAGE gels were scanned and quantified by using the ChemiDoc MP imaging system (Bio-Rad) and ImageJ. The intensity of the displayed S-trimer protein at various time points was compared to that at the time zero to assess any reduction in the amount of intact Spike protein associated with phage.

The vaccine receptor-binding functionality at various time points was assessed by the ability of the displayed S-trimers to bind to human ACE2 receptor, as previously described (20). Briefly, 1 × 10^10^ vaccine or control phages were coated on ELISA plates overnight at 4°C, followed by blocking with PBS–5% bovine serum albumin (BSA) buffer at 37°C for 1 h. The human ACE2-mouse Fc fusion protein (Sino Biological) was added, followed by incubation for 1 h at 37°C. Then, the secondary goat anti-mouse IgG-HRP antibody was added, followed by incubation for 1 h at 37°C. The plates were developed with TMB (tetramethylbenzidine) substrate and stop buffer, and the absorbance was measured at 650 nm on a VersaMax spectrophotometer.

### Beta-S-trimer (tag-free) purification.

To obtain prefusion-stabilized native-like trimers, Secto or Secto-β trimers were expressed from a recombinant plasmid in ExpiCHO mammalian host cells. The CHO cell growth and Spike recombinant plasmid transfection were performed according to the ExpiCHO expression system User Guide (MAN0014337; Thermo Fisher). S-trimer expression was under the control of a strong cytomegalovirus promoter. Cultures were harvested 8 days after transfection by centrifuging the cells at 3,000 × *g* for 20 min at 4°C. The supernatant (culture medium) containing the expressed S-trimers was recovered and clarified through a 0.22-μm-pore-size filter (Corning, Inc.) for column purification.

The pH of the filtered supernatant (250 mL) was first adjusted to 8 using 1 M Tris-HCl (pH 8). Then the supernatant was loaded onto two Hi-TRAP Q-FF columns connected in tandem and previously equilibrated with wash buffer (100 mM NaCl, 50 mM Tris-HCl [pH 8]). The sample was loaded at a flow rate of 1 mL/min using the AKTA Prime-Plus liquid chromatography system (GE Healthcare). The flowthrough was collected and diluted with 50 mM Tris-HCl (pH 8) buffer at a 1:1 ratio and loaded onto the Hi-TRAP Q-HP column at a flow rate of 1 mL/min, followed by washing the column with 50 mM NaCl–50 mM Tris-HCl (pH 8) wash buffer until the absorbance reached the baseline. The trimers were eluted using a 50 to 600 mM linear gradient of salt in 50 mM Tris-HCl (pH 8; 90 mL total gradient). The peak fractions were run on a 4 to 20% SDS-PAGE gradient to select fractions with a high ratio of trimers to contaminants. The selected fractions were then pooled and concentrated using 100-kDa filters (Millipore) and loaded onto a Hi-Load 16/600 Superdex-200 pg (preparation grade) size-exclusion chromatography column (GE Healthcare) equilibrated with gel filtration buffer (100 mM NaCl, 50 mM Tris-HCl [pH 8]) to further separate the low-molecular-weight contaminants and obtain purified trimers (ÄKTA FPLC; GE Healthcare). Eluted trimer fractions were assessed on an SDS-PAGE gel to determine the purity, and selected fractions were pooled and passed through 0.22-μm-pore-size filter to sterilize the sample. If needed, the trimers were concentrated using 100-kDa centrifugal filters at 3,500 rpm in a swinging-bucket rotor. The concentration of the Secto trimers was kept around 1 to 2 mg/mL. Protein aliquots (1 mL size) were made, flash-frozen in liquid nitrogen, and stored at −80°C until use.

### Mouse immunizations.

We followed the recommendations of the National Institutes of Health for mouse studies (*Guide for the Care and Use of Laboratory Animals*). All animal experiments were approved by the Institutional Animal Care and Use Committee of the Catholic University of America (Washington, DC; Office of Laboratory Animal Welfare assurance number A4431-01) and the University of Texas Medical Branch (Galveston, TX; Office of Laboratory Animal Welfare assurance number A3314-01). The SARS-CoV-2 virus challenge studies were conducted in the animal BSL-3 (ABSL-3) suite at UTMB. Five-week-old female BALB/c (Jackson Laboratory) or hACE2-transgenic AC70 (Taconic Biosciences) mice were randomly grouped (5 to 10 animals per group) and allowed to acclimate for 14 days. The phage T4-CoV-2 vaccine was administered either i.m. or i.n. into the hind legs or nares of mice, respectively. For two-dose regimen, animals received vaccination at days 0 (prime) and 21 (boost), while for one-dose regimen, the vaccine was administered at day 21. Three different number of phage particles possessing 0.8, 4.8, and 20 μg of S-trimer antigens representing ~1.0 × 10^10^, 6 × 10^10^, and 2.5 × 10^11^ phage particles, respectively, were used. Negative-control mice received the same volume of PBS or the same amount of T4 control phage (T4 control). Blood was drawn from each animal on day 0 (prebleed) and day 42, and the isolated sera were stored at −80°C until further use.

### Bronchoalveolar lavage fluid collection.

On day 21 after boosting, bronchoalveolar lavage fluid (BALF) samples were obtained from immunized and control animals according to a previously described protocol (83), with slight modifications. Briefly, the salivary glands were dissected to expose the tracheas of euthanized mice (*n* = 5/group). A small incision was made on the ventral face of the trachea, and a blunt 26G needle was inserted into the trachea and secured by tying the trachea around the catheter using the floss placed underneath the trachea. An aliquot (600 μL) of PBS loaded into a 1-mL syringe was flushed in the lungs, and BALF specimens were collected.

### ELISA determination of IgG, IgG subtypes, and IgA antibodies.

ELISA plates (Evergreen Scientific) were coated with 100 μL (1 μg/mL) per well of SARS-CoV-2 Secto protein (Sino Biological), SARS-CoV-2 Secto-β protein, SARS-CoV-2 RBD-untagged protein (Sino Biological), SARS-CoV-2 NP (Sino Biological), or SARS-CoV-2 E protein (1 to 75 amino acids; Thermo Fisher Scientific) in coating buffer (0.05 M sodium carbonate-sodium bicarbonate [pH 9.6]) at 4°C for overnight incubation. The plates were washed twice with PBS buffer, followed by blocking with 200 μL per well of PBS (pH 7.4)–5% BSA buffer at 37°C for 2 h. Serum and BALF samples were diluted with a 5-fold dilution series beginning with an initial 100-fold dilution in PBS–1% BSA. Then, 100 μL of diluted serum or BALF samples was added to each well, and the plates were incubated at 37°C for 1 h. The plates were washed five times with PBST (PBS + 0.05% Tween 20). Next, the secondary antibody was added at a 1:10,000 dilution in PBS–1% BSA buffer (100 μL/well) using either goat anti-mouse IgG-HRP, goat anti-mouse IgG1-HRP, goat anti-mouse IgG2a-HRP, or goat anti-mouse IgA-HRP (Thermo Fisher Scientific). After incubation for 1 h at 37°C and five washes with PBST buffer, the plates were developed using the TMB (3,3′,5,5′-tetramethylbenzidine) Microwell peroxidase substrate system (KPL; 100 μL) for 5 to 10 min. The enzymatic reaction was stopped by adding 100 μL of TMB BlueSTOP solution (KPL). The absorbance of the optical density at 650 nm was read within 30 min on a VersaMax spectrophotometer. The endpoint titer was defined as the highest reciprocal dilution of serum that gives an absorbance >2-fold of the mean background of the assay.

### Virus neutralization assay.

Neutralizing antibody titers in mouse immune sera against SARS-CoV-2 US-WA-1/2020 or its Beta, Delta, or Omicron variants were quantified by using Vero E6 cell-based microneutralization assay in the BSL-3 suite, as previously described (20). Briefly, serially 1:2 or 1:3 downward-diluted mouse sera (original dilutions, 1:10 or 1:20) that were decomplemented at 56°C for 60 min in a 60 μL-volume were incubated for 1 h at room temperature in duplicate wells of 96-well microtiter plates that contained 120 infectious SARS-CoV-2 virus particles in 60 μL in each well. After incubation, 100 μL of the mixture in individual wells was transferred to a Vero E6 cell monolayer grown in 96-well microtiter plates containing 100 μL of minimal essential medium/2% fetal bovine serum (FBS) medium in each well and then cultured for 72 h at 37°C before we determined the presence or absence of cytopathic effect (CPE). Neutralizing antibody titers of the tested specimens were calculated as the reciprocals of the highest dilution of sera that completely inhibited virus-induced CPE.

### T cell proliferation, phenotypes, and cytokine analysis.

To measure T cell proliferation, a bromodeoxyuridine (BrdU; a thymidine analog) incorporation method was used. Briefly, spleens were aseptically removed from five animals in each indicated group on day 21 after the last immunization dose. Spleens were homogenized and passed through a 70-μm-pore size cell strainer to obtain single cell suspension in RPMI 1640 cell culture medium. Splenocytes were then seeded into 24-well tissue culture plates at a density of 2.0 × 10^6^ cells/well (four wells/mouse) and stimulated with either SARS-CoV-2 S-trimer (10 to 100 μg/mL) or SARS-CoV-2 PepTivator Peptide S and NP protein pools (10 μg/mL each, Miltenyi Biotec) for 72 h at 37°C. BrdU (BD Bioscience) was added to a final concentration of 10 μM during the last 18 h of incubation with the stimulants to be incorporated into the splenocytes. Subsequently, the BrdU-labeled splenocytes were surface stained for T-cell (CD3e-APC; eBioscience) marker after blocking with anti-mouse CD16/32 antibodies (BioLegend). The cells were then permeabilized and treated with DNase to expose BrdU epitopes, followed by anti-BrdU-FITC and 7-AAD (7-amino-actinomycin D) staining using a BD Pharmingen FITC BrdU flow kit. The splenocytes were then subjected to flow cytometry, and data were analyzed as we previously described (84, 85). The percentages of BrdU^+^ cells in CD3^+^ populations were calculated using FACSDiva software.

To measure T cell phenotypes, the overnight (16 h)-stimulated splenocytes described above were similarly blocked with anti-mouse CD16/32 antibodies (BioLegend) and stained with Fixable Viability Dye eFluor 506 (eBioscience), followed by APC/anti-mouse CD3e (eBioscience), PE/Dazzle 594 anti-mouse CD4 (BioLegend), and FITC/anti-mouse CD8 (BioLegend) for CD3, CD4, and CD8 T cell surface markers, respectively. The cells were then permeabilized for intracellular staining with PerCP/Cy5.5 anti-mouse IFN-γ, PE/Cy7 anti-mouse IL-17A (BioLegend), eFluor 450/anti-mouse TNF-α (eBioscience) and analyzed by flow cytometry.

To assess cytokine production, cell supernatants were collected after stimulation with S-trimers as described above for 72 h at 37°C. Cytokines in the supernatants were then measured using a Bio-Plex Pro mouse cytokine 23-plex assay (Bio-Rad Laboratories). Likewise, BALF from control and immunized mice was used to measure cytokines.

### 16S rRNA gene sequencing and microbiome analysis.

Fecal pellets were collected from five animals in each indicated group on day 21 after the last immunization dose. Total genomic DNA was extracted from the fecal matter using methods previously described (86). DNA samples were further purified using a DNA Clean & Concentrator kit (Zymo Research).

The extracted microbial DNA described above was then subjected to amplification and sequencing of the V4 region of the 16S rRNA gene by using a NEXTflex 16S V4 Amplicon Seq kit 2.0 (Perkin-Elmer), and sequences were generated on an Illumina MiSeq platform (Illumina). Raw reads were filtered using the Lotus pipeline, followed by *de novo* clustering to operational taxonomic units (OTU) at 97% sequence identity with UPARSE. Bacterial diversity and community composition were evaluated using QIIME v1.8, and taxonomy assignment of the representative sequence for each OTU was completed using the RDP classifier algorithm and the SILVA reference database (v123) (87).

### Animal challenges.

Immunized and control mice were first ear tagged and their initial weights recorded. Mice were then anesthetized and i.n. challenged with 60 μL of either SARS-CoV-2 MA10 strain for conventional mice or SARS-CoV-2 US-WA-1/2020 strain or the Delta variant (B.1.617.2) for hACE2-transgenic mice. The challenge dose was ~10^5^ TCID_50_. For hACE2-transgenic mice, the challenge dose was 300 TCID_50_. The animals were monitored for the onset of morbidity (weight loss and other signs of illness, every day) and mortality over the indicated period.

### Histopathology studies.

Lung tissues were excised from euthanized animals (immunized and control) at 2 to 5 days postchallenge and immersion fixed in 10% neutral buffered formalin. After fixation, tissues were sectioned at 5 μm, mounted on glass slides, and stained with H&E and MOVAT for histopathological analysis (Department of Pathology, UTMB). Staining with MOVAT helps to better visualize tissue architecture. Histopathological analysis of lung sections from BALB/c mice was performed based on three parameters: mononuclear inflammatory infiltrate around bronchovascular bundles, interstitial inflammation, and alveolar exudate/hemorrhage. Scores for bronchovascular infiltrates ranged from 0 (normal) to 3, as follows: 1, occasional mononuclear infiltrates, 5 to 10 μm thick; 2, multifocal mononuclear infiltrates, 5 to 20 μm thick; and 3, diffuse mononuclear infiltrates, >20 μm thick. The scores for interstitial inflammation were as follows: 1, occasional areas of widened alveolar septa; 2, multifocal areas of widened alveolar septa; and 3, diffused widening of alveolar septa. For alveolar exudate/hemorrhage, the scores were as follows: 1, occasional areas of alveolar exudate/hemorrhage; 2, multifocal areas of alveolar exudate/hemorrhage; and 3, diffused areas of alveolar exudate/hemorrhage. The combined scores for the vector control group and the T4-CoV-2 vaccine group were analyzed by using a Student *t* test.

For hACE2-transgenic mice, histopathological analysis was performed based on the following parameters: interstitial inflammation/alveolar exudate and mononuclear inflammatory infiltrate around bronchovascular (BV) bundles. Interstitial inflammation/alveolar exudates were scored based on the percentage of lung surface area involved (0 to 100%), while scores for BV infiltrate ranged from 0 (normal) to 3 as follows: 1, occasional mononuclear infiltrates, 5 to 10 μm thick; 2, multifocal mononuclear infiltrates, 5 to 20 μm thick; and 3, diffused mononuclear infiltrates, >20 μm thick. The scores for the intranasal PBS control group, the T4 vector control group, and the T4-CoV-2-vaccinated group were analyzed by using a Student *t* test if the groups passed the normality test (Shapiro-Wilk) or a Mann-Whitney rank sum test if the normality test failed.

### Viral load determination.

For virus quantitation, the remaining portion of the lungs were weighed and frozen at −80°C. Thawed lungs were homogenized in PBS–10% FBS solution using a TissueLyser (Qiagen, Retsch, Germany). The homogenates were centrifuged, and SARS-CoV-2 titers in the clarified fluids were determined by serial dilution in quadruplicate wells of Vero E6 cells in 96-well plates. Titers of virus in lung homogenates were expressed as TCID_50_/g of lung (log_10_) as we previously described (88).

### Photo credit.

The mouse and immune cell images were created with BioRender.com. The figure data were organized by Photoshop CS6 (Adobe).

### Statistics and software.

Statistical analyses were performed by GraphPad Prism 9.0 software by using one-way or two-way analysis of variance (ANOVA) with Tukey’s *post hoc* test or multiple t-test according to the generated data. We used Kaplan-Meier with log-rank (Mantel-Cox) test for animal survival studies. Significant differences between two groups are indicated by asterisks (*, *P* < 0.05; **, *P* < 0.01; ***, *P* < 0.001; ****, *P* < 0.0001) in the figures (“ns” indicates not significant).

### Data availability.

All data are available in the main text or the supplemental materials.
