# Supplementary figures and images for: A Bacteriophage-Based, Highly Efficacious, Needle- and Adjuvant-Free, Mucosal COVID-19 Vaccine
Source: mBio. 2022 Jul 28;13(4):e01822-22. doi: 10.1128/mbio.01822-22 (PMC9426593; doi:10.1128/mbio.01822-22)

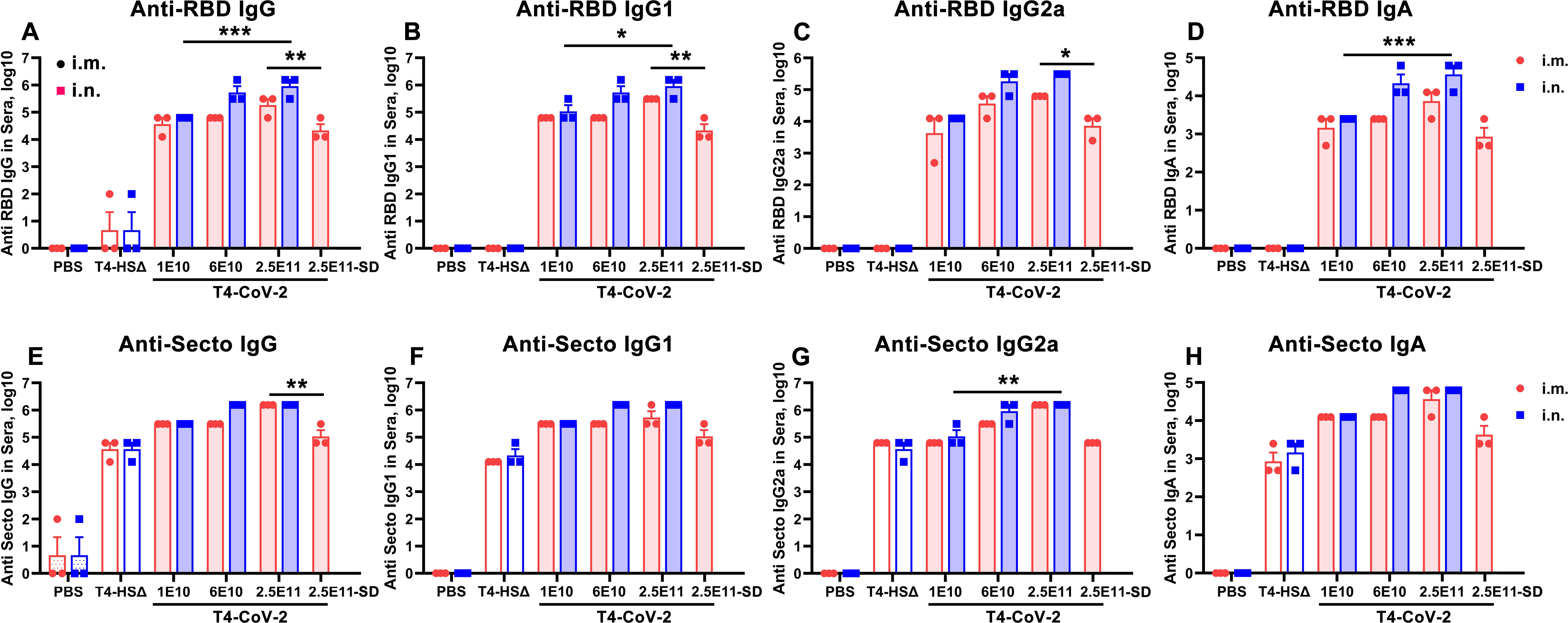

Supplement: FIG S1 [file mbio.01822-22-s0001.tif]

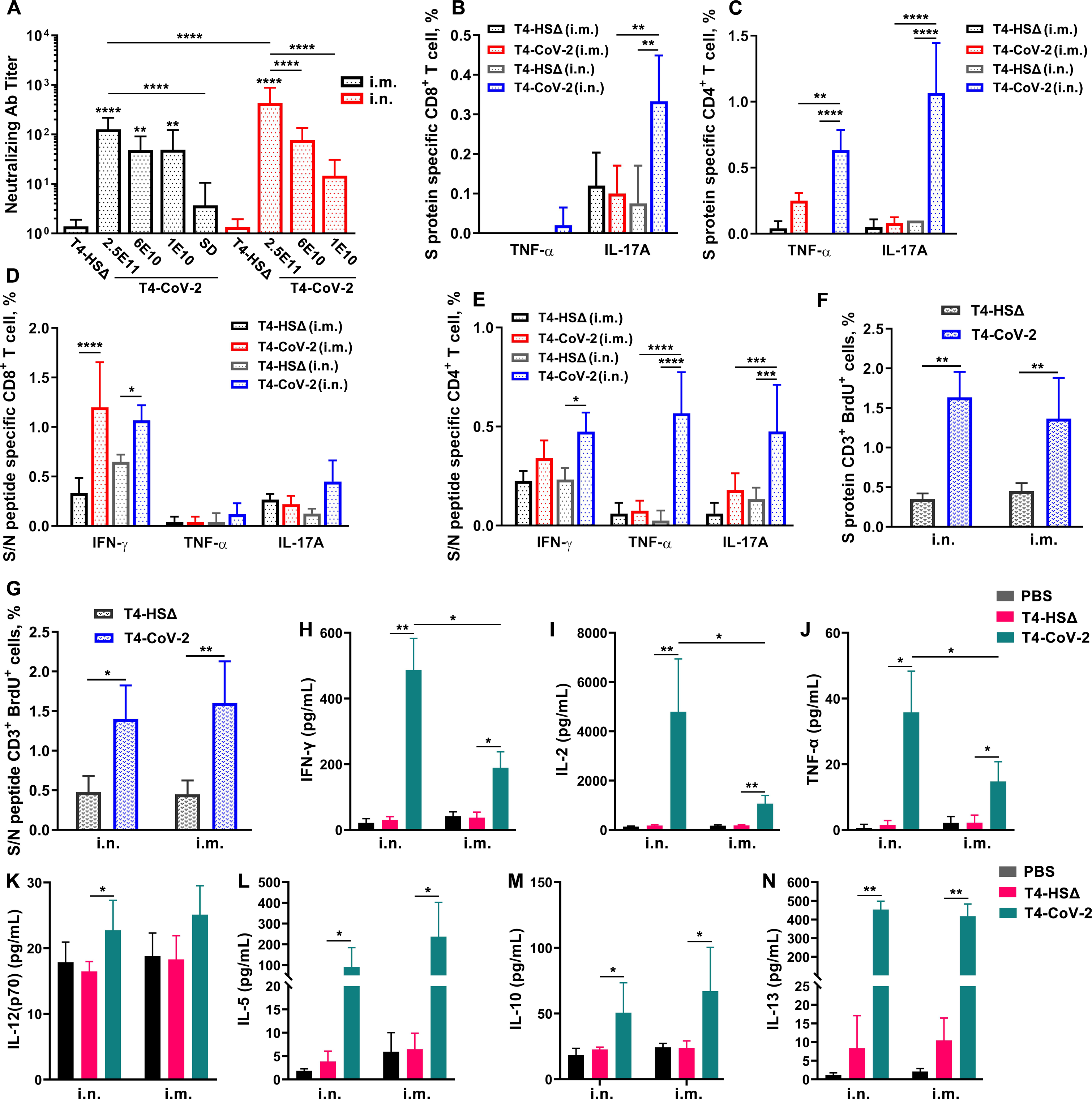

Supplement: FIG S2 [file mbio.01822-22-s0002.tif]

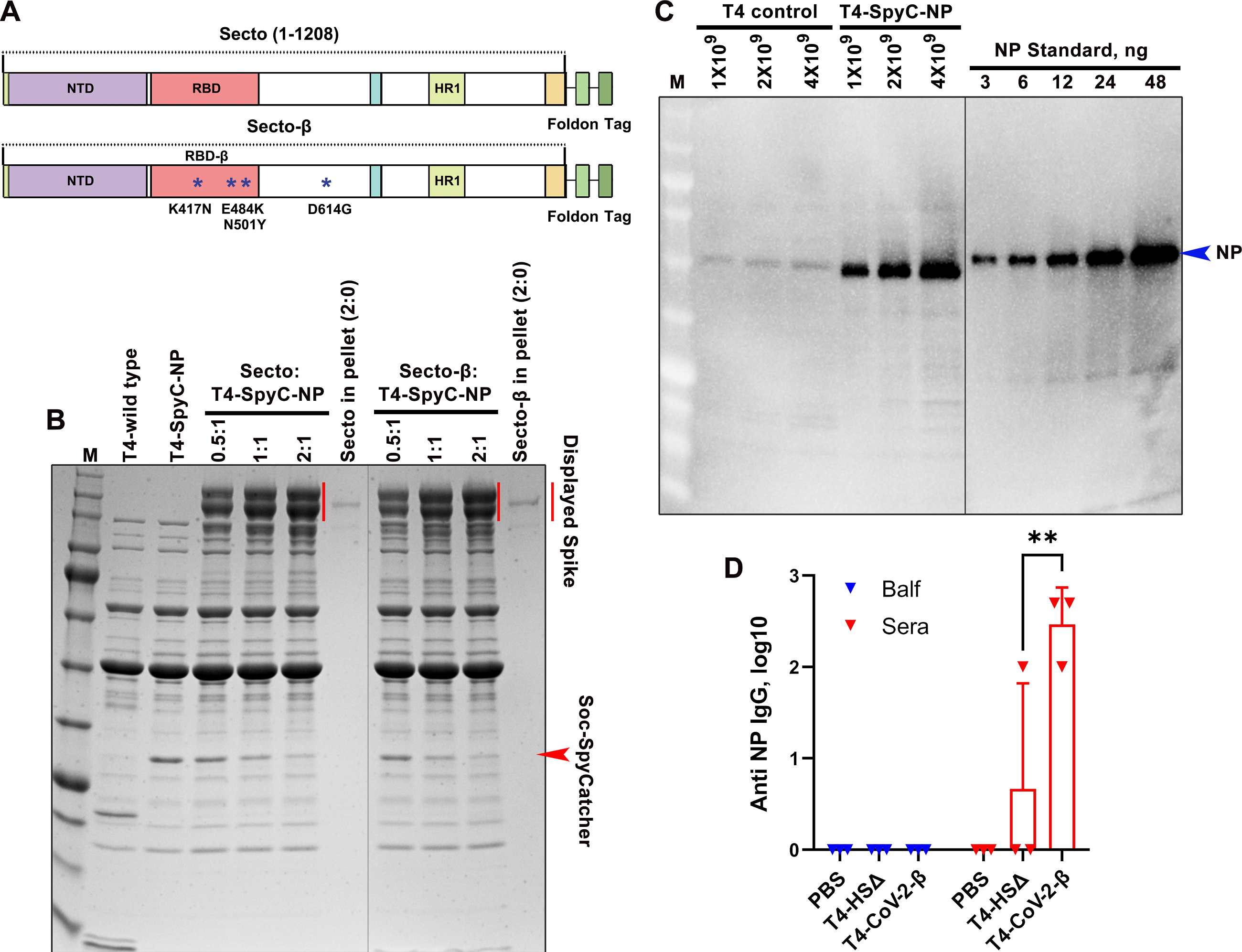

Supplement: FIG S3 [file mbio.01822-22-s0003.tif]

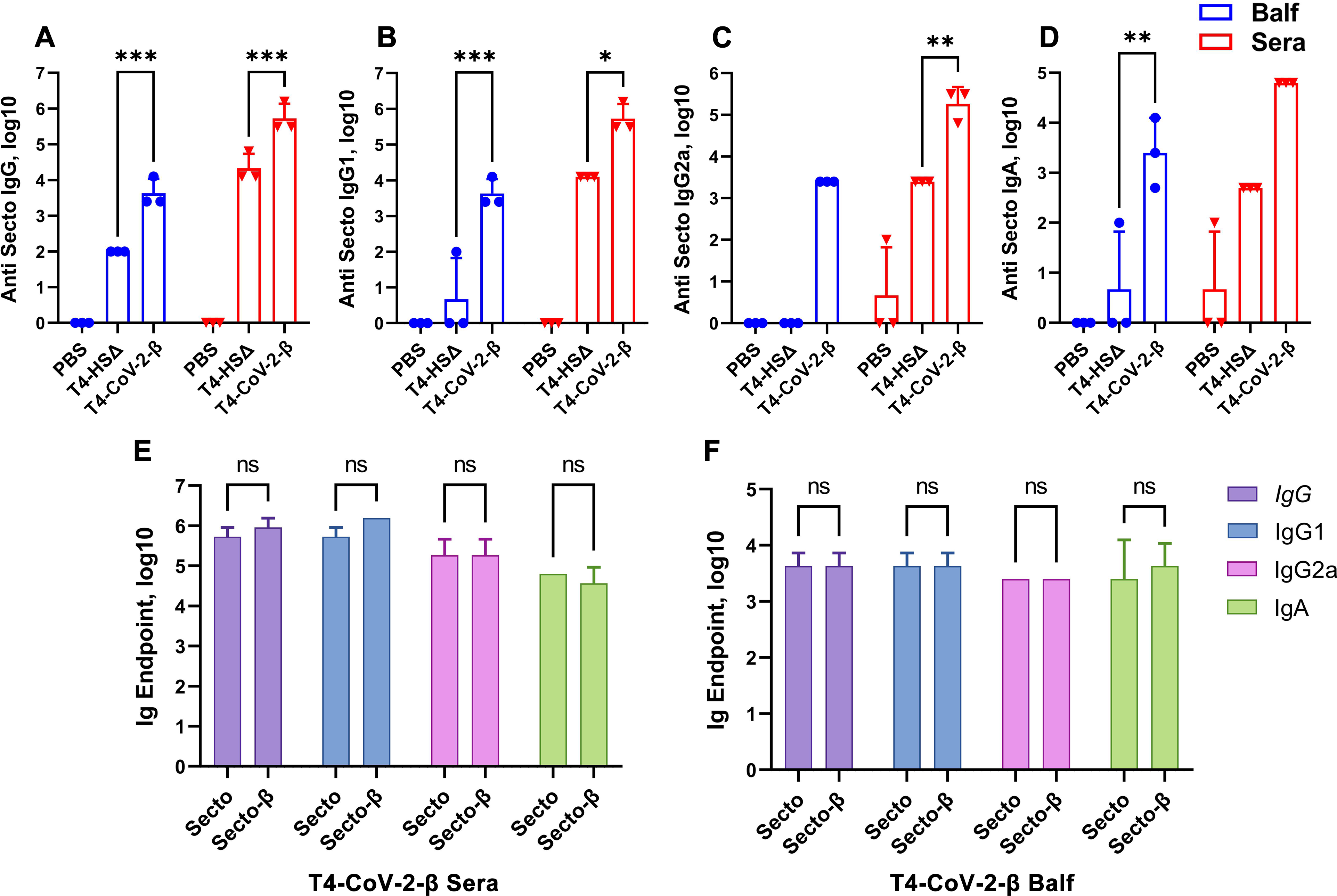

Supplement: FIG S4 [file mbio.01822-22-s0004.tif]

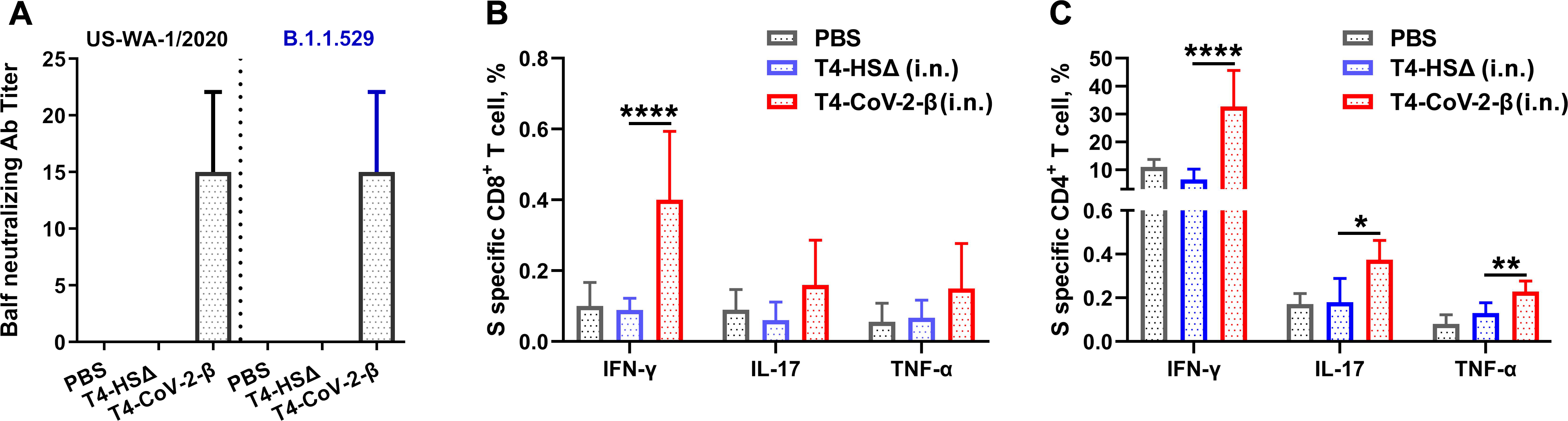

Supplement: FIG S5 [file mbio.01822-22-s0005.tif]
